# Joint Analysis of Phenotypic and Genomic Diversity Sheds Light on the Evolution of Xenobiotic Metabolism in Humans

**DOI:** 10.1093/gbe/evac167

**Published:** 2022-11-29

**Authors:** Médéric Mouterde, Youssef Daali, Victoria Rollason, Martina Čížková, Anwar Mulugeta, Khalid A Al Balushi, Giannoulis Fakis, Theodoros C Constantinidis, Khalid Al-Thihli, Marie Černá, Eyasu Makonnen, Sotiria Boukouvala, Said Al-Yahyaee, Getnet Yimer, Viktor Černý, Jules Desmeules, Estella S Poloni

**Affiliations:** Department of Genetics and Evolution (GENEV), University of Geneva, Geneva, Switzerland; Division of Clinical Pharmacology and Toxicology, Geneva University Hospitals and University of Geneva, Geneva, Switzerland; Division of Clinical Pharmacology and Toxicology, Geneva University Hospitals and University of Geneva, Geneva, Switzerland; Institute of Archaeology of the Academy of Sciences of the Czech Republic, Prague, Czech Republic; Department of Pharmacology and Clinical Pharmacy, College of Health Sciences, Addis Ababa University, Addis Ababa, Ethiopia; College of Pharmacy, National University of Science and Technology, Muscat, Sultanate of Oman; Department of Molecular Biology and Genetics, Democritus University of Thrace, Alexandroupolis, Greece; Department of Medicine, Democritus University of Thrace Medical School, Alexandroupolis, Greece; Department of Genetics, Sultan Qaboos University Hospital, Muscat, Sultanate of Oman; Department of Medical Genetics, Third Faculty of Medicine, Charles University, Prague, Czech Republic; Department of Pharmacology and Clinical Pharmacy, College of Health Sciences, Addis Ababa University, Addis Ababa, Ethiopia; Center for Innovative Drug Development and Therapeutic Trials for Africa, College of Health Sciences, Addis Ababa University, Addis Ababa, Ethiopia; Department of Molecular Biology and Genetics, Democritus University of Thrace, Alexandroupolis, Greece; Department of Genetics, College of Medicine and Health Sciences, Sultan Qaboos University, Muscat, Sultanate of Oman; Center for Global Genomics & Health Equity, Department of Genetics, Perelman School of Medicine, University of Pennsylvania, Philadelphia, Pennsylvania, USA; Department of Anthropology and Human Genetics, Faculty of Science, Charles University, Prague, Czech Republic; Division of Clinical Pharmacology and Toxicology, Geneva University Hospitals and University of Geneva, Geneva, Switzerland; Department of Genetics and Evolution (GENEV), University of Geneva, Geneva, Switzerland; Institute of Genetics and Genomics of Geneva (iGE3), Geneva, Switzerland

**Keywords:** ADME genes, drug metabolism phenotypes, genome-wide association studies, genome-wide selection scans, human evolution

## Abstract

Variation in genes involved in the absorption, distribution, metabolism, and excretion of drugs (ADME) can influence individual response to a therapeutic treatment. The study of ADME genetic diversity in human populations has led to evolutionary hypotheses of adaptation to distinct chemical environments. Population differentiation in measured drug metabolism phenotypes is, however, scarcely documented, often indirectly estimated via genotype-predicted phenotypes. We administered seven probe compounds devised to target six cytochrome P450 enzymes and the P-glycoprotein (P-gp) activity to assess phenotypic variation in four populations along a latitudinal transect spanning over Africa, the Middle East, and Europe (349 healthy Ethiopian, Omani, Greek, and Czech volunteers). We demonstrate significant population differentiation for all phenotypes except the one measuring CYP2D6 activity. Genome-wide association studies (GWAS) evidenced that the variability of phenotypes measuring CYP2B6, CYP2C9, CYP2C19, and CYP2D6 activity was associated with genetic variants linked to the corresponding encoding genes, and additional genes for the latter three. Instead, GWAS did not indicate any association between genetic diversity and the phenotypes measuring CYP1A2, CYP3A4, and P-gp activity. Genome scans of selection highlighted multiple candidate regions, a few of which included ADME genes, but none overlapped with the GWAS candidates. Our results suggest that different mechanisms have been shaping the evolution of these phenotypes, including phenotypic plasticity, and possibly some form of balancing selection. We discuss how these contrasting results highlight the diverse evolutionary trajectories of ADME genes and proteins, consistent with the wide spectrum of both endogenous and exogenous molecules that are their substrates.

**Significance**

Physicians often observe that their patients can react differently to the same medical treatment: for some patients, the drug will prove inefficient, whereas for others, it might provoke side effects, sometimes rather serious. These differences in response to exogenous compounds are phenotypic differences that can be due to several factors, including genetic factors, potentially unevenly distributed among human populations. Using a standardized protocol, we demonstrate significant variation among Ethiopians, Omanis, Greeks, and Czechs in six out of seven measured phenotypes linked to 70–80% of drugs commonly used in the clinic. Significant genetic associations are evidenced by GWAS for four of these phenotypes, but genome scans of selection fail to detect positive signals in the genetic loci identified. We hypothesize that the results of our joint analysis of phenotypic and genomic diversity are best explained by diverse mechanisms, including phenotypic plasticity and possibly balancing selection, but do not appear to favor positive selection as an adaptation to specific chemical environments.

## Introduction

Among the numerous factors that influence the outcome of a treatment, including a patient's health condition and life-history traits (Evrard and Mbatchi, 2012), it is recognized that specific genotypes can also contribute in a significant way (Meyer, 2004). The last three decades have witnessed intense interest in the predictability of response to medication that can be attributed to the genetic makeup of patients, due to its potential to develop clinical biomarkers to ensure the efficacy and safety of existing and new drugs (Lu et al. 2014; Pirmohamed, 2014; Kalman et al. 2016). For instance, this interest has led the industry-initiated initiative PharmaADME Working Group (http://pharmaadme.org) to publish a list of “evidence-based ADME genes”, that is polymorphic genes known to be involved in the differential absorption, distribution, metabolism, and excretion of drugs (Huang et al. 2006; Sim et al. 2011; Tilleman et al. 2019). Long-recognized examples of these “pharmacogenes” include the highly polymorphic *CYP2D6* and *NAT2* genes (Meyer, 2004), both of which display significantly variable allelic distributions among human populations (Patin et al. 2006; Sistonen et al. 2007; Sabbagh et al. 2008, 2011, 2018; Fuselli et al. 2010; Mortensen et al. 2011; LLerena et al. 2014; Podgorná et al. 2015).

More recently, the development of high-throughput genotyping and sequencing technologies has allowed the performance of genome-wide association studies (GWAS) that screen entire genomes in relation to drug-related phenotypes, such as metabolism rate or side effects (Daly, 2012; Giacomini et al. 2017). One successful example of the potential of this approach was the identification of single nucleotide polymorphism (SNP) rs12979860 near gene *IFNL3*, associated with a significant change in response to the formerly widespread interferon-based anti-hepatitis C virus treatment (Ge et al. 2009; Lu et al. 2014). Similar to polymorphisms linked to *CYP2D6* and *NAT2*, substantial frequency variation among populations has been shown for rs12979860 at the worldwide scale (Thomas et al. 2009). However, as promising as the GWAS approach was first expected to be, it was subsequently challenged by the extent of rare deleterious variation identified in human genomes (Nelson et al. 2012), and the likely small but combined effects of these genetic variants on disease or drug-response phenotypes (Dugger et al. 2018). Moreover, environmental factors which are known to influence drug-related phenotypes, such as drug-drug interactions, drug-food interactions, and disease, can lead to uncorrelated genotype–phenotype data.

Most of the already identified ADME genes display significant population variation, which is indicative of the existence of common variants with differentiated population frequency distributions (Torkamani et al. 2012; Fuselli, 2019). The structure of this diversity at most of these loci mirrors that observed for the rest of the genome, thus underlining the role of human demographic history in the build-up of this diversity (Sistonen et al. 2009; Delser and Fuselli, 2013). However, along with neutral processes shaping genomic diversity, adaptive processes to respond to specific chemical or dietary environments have also been evidenced for several genes (Fan et al. 2016; Vangenot et al. 2019), among which are genes of the immune system in relation to differential pathogenic environments or episodes (Sanchez-Mazas, 2020). Because of their functions at the interface between the environment and the organism, some ADME genes are also possible targets of adaptive processes in response to selective pressures at the local (for instance, a local environmental component of the diet, as shown for arsenic concentrations in drinking water, Schlebusch et al. 2015) or global levels (for instance, it is speculated that the consumption of fermented foods, a cultural process shared among human diets, could exert some selective pressure, Amato et al. 2021, Dunn et al. 2021). Indeed, environmental adaptation has been documented for several genes of the cytochrome P450 system (commonly referred to as CYP genes), a family of highly polymorphic ADME genes known for their role in the detoxification or metabolism of exogenous compounds, and also generally involved in several endogenous metabolic pathways (Fuselli, 2019). Interestingly, the vast majority (around 70% to 80%) of drugs commonly used in the clinic are metabolized by less than 10 of these CYP enzymes (Zanger and Schwab, 2013), thereby motivating the development of phenotyping cocktails simultaneously targeting the activity of these genes to predict a patient's response to a specific chemical therapy (Pirmohamed, 2014). If a genetically determined activity is modulated by environmental factors (Zanger and Schwab, 2013; Aklillu, 2018), GWAS analysis of the phenotypes obtained with a cocktail approach has the potential to determine if the influence of genetic factors on phenotype is relevant in comparison to environmental factors. Moreover, it can also reveal unexpected genetic contributions to specific phenotypes.

Although studies on world-wide populations are becoming more frequent, some regions are still underrepresented, notably the African continent and the Middle East, contrary to European and East Asian populations (LLerena et al. 2014; Fricke-Galindo et al. 2016). Moreover, outside of the clinical context, studies comparing phenotype distributions determined on the basis of actual drug-related metabolic activities in different populations still remain uncommon. Indeed, most studies are designed as case/control clinical investigations, thus limiting the generalization of results to the general healthy population. In addition, present knowledge is rather dominated by so-called “genetic phenotypes”, namely phenotype frequency distributions predicted from genotyping. Therefore, measuring metabolic phenotypes in diverse population samples is necessary to assess the extent of phenotypic differences between populations (Fricke-Galindo et al. 2016). This can be addressed using a standardized phenotyping protocol in diverse healthy populations.

**Fig. 1.**
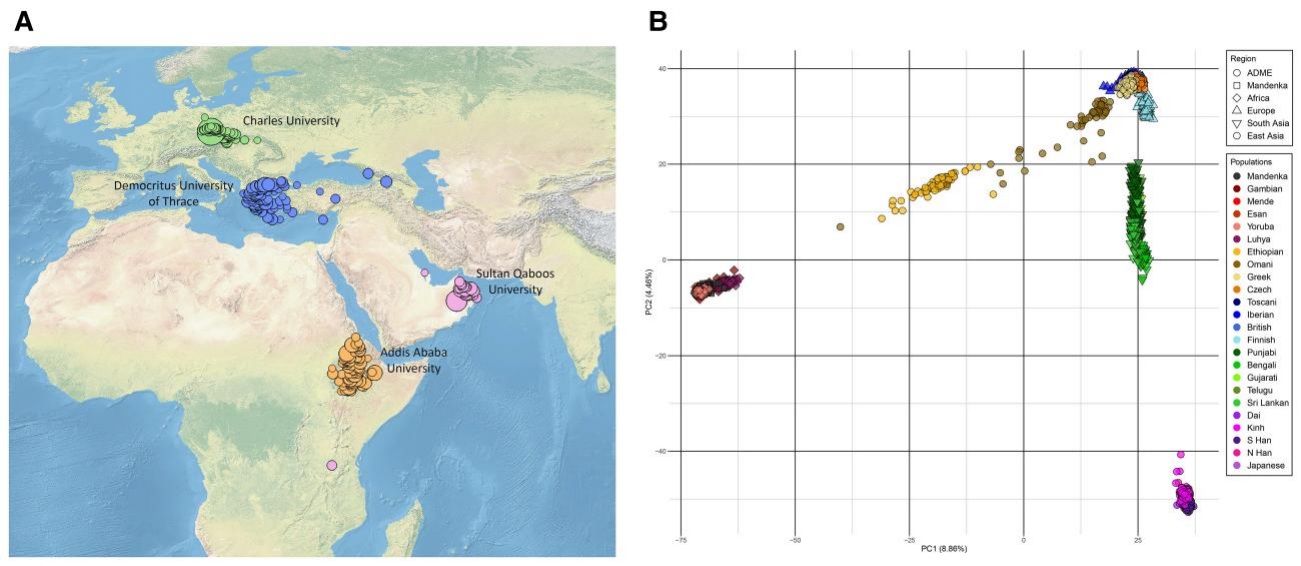
Birthplaces of the participants’ grandparents and PCA comparison to 1KG population samples from Africa, Europe, and Asia. (*A*) Frequencies of birthplaces of the four grandparents of all 367 participants to the study, with an indication of sampling sites. (*B*) Principal component analysis of the four ADME, the Senegalese Mandenka, and the 19 1KG population samples. The ADME dataset is represented by circles. The first and second components account for 8.86% and 4.46% of the total variance, respectively.

In this study, by implementing a safe and efficient protocol to both collect individual DNA and measure blood concentrations of six probe drugs and their specific metabolites, as well as the P-glycoprotein (P-gp) substrate concentration in the same individuals, we jointly undertook phenotyping and genotyping of four human populations located along a latitudinal transect from Africa to the Middle East and Europe, that is Ethiopians, Omanis, Greeks, and Czechs (fig. 1). Each population was represented by a large sample of healthy young volunteers (from 56 to 102 individuals per population successfully genotyped and phenotyped), without any medication or pathological conditions, so as to perform a GWAS for each of the tested probe compounds. The protocol included the Geneva phenotyping cocktail, composed of seven probe drugs (caffeine, bupropion, flurbiprofen, omeprazole, dextromethorphan, midazolam, and fexofenadine), which is routinely used in the clinical context to measure the activity of six CYP enzymes (CYP1A2, CYP2B6, CYP2C9, CYP2C19, CYP2D6, and CYP3A4) and the P-gp transporter (Bosilkovska et al. 2014a; Rollason et al. 2020). We analyzed this newly generated data so as to estimate the extent of phenotypic differentiation between human populations in their capacity to metabolize drugs, and to assess a genetic contribution to this variation through GWAS. Using the most differentiated populations in phenotypic distribution, we compared the GWAS signals to those outputted by genome scans of selection. We discuss our results in the light of present knowledge and assumptions on evolutionary mechanisms acting on specific ADME genes.

## Results

### Phenotypic Comparisons

Using the concentrations of both substrates and their metabolites measured at three time points after probe drugs administration with the Geneva cocktail, we calculated areas under the curve (AUC) and defined an individual phenotype (i.e., its level of enzymatic activity) as the AUC_metabolite_/AUC_substrate_ ratio, except for the transporter activity of P-gp, which was measured directly by the AUC_substrate_, that is the P-gp probe drug concentration (fexofenadine). Hence, higher ratios correspond to faster enzymatic activities, except for fexofenadine, for which a higher AUC_substrate_ corresponds to a slower drug transporter activity.

**Fig. 2.**
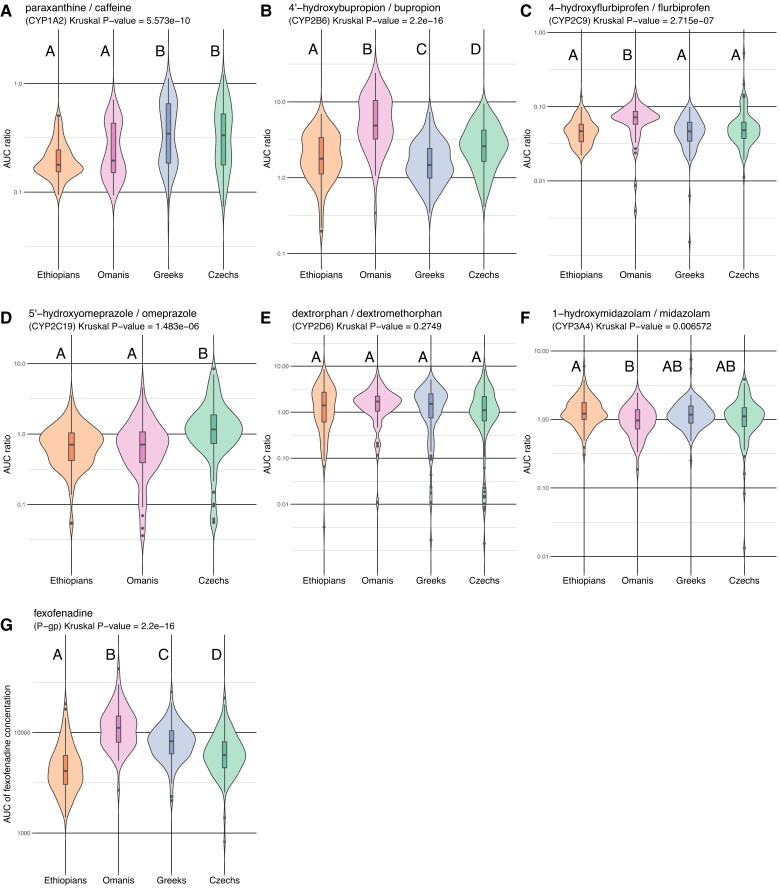
Violin plots of AUC ratios of the seven compounds of the Geneva cocktail in Ethiopians, Omanis, Greeks, and Czechs. (*A*) Caffeine. (*B*) Bupropion. (*C*) Flurbiprofen. (*D*) Omeprazole. (*E*) Dextromethorphan. (*F*) Midazolam. (*G*) Fexofenadine. AUC ratios are displayed in a logarithmic scale. For fexofenadine (panel *G*), only AUC is reported (see text). For each phenotype, the figure reports the *P-*value of the Kruskal–Wallis homogeneity test. Upper case letters on the top of violin plots (A, B, C, D, AB) indicate significantly differentiated groups according to pairwise Wilcoxon tests (see supplementary table S1, Supplementary Material online). The AB group in panel F (midazolam/CYP3A4) is undifferentiated from both groups A and B.

Kruskal–Wallis and Wilcoxon two-by-two tests, to compare the AUC substrate/metabolite ratios for the Geneva cocktail compounds among populations, yielded differentiated results (fig. 2, supplementary table S1, Supplementary Material online). Out of the seven compounds tested, six have associated phenotype distributions differing in at least one population. This pattern is more pronounced for probe drugs bupropion (CYP2B6) and fexofenadine (P-gp), for which the average measured enzymatic or transporter activities are significantly differentiated between all four populations. The highest CYP2B6 average metabolic rate was observed in the Omani population and the lowest in the Greeks. The Omanis displayed the slowest transport activity for P-gp, with the fastest observed in Ethiopians. The enzymatic activity measured for probe drug caffeine (CYP1A2) separates the populations into two significantly differentiated groups, the two European populations displaying higher average metabolic rates than Ethiopians and Omanis. A similar pattern was observed for probe drug omeprazole (CYP2C19), with Czechs being significantly faster, on average, than Ethiopians and Omanis. However, due to an omeprazole supply problem, the Greek sample was not phenotyped with this probe drug. The enzymatic activities measured for probe drug flurbiprofen (CYP2C9) were found statistically undifferentiated in three of the four populations tested, while significantly faster, on average, in Omanis. In turn, a significantly lower average rate was found in Omanis and a higher one in Ethiopians for probe drug midazolam (CYP3A4), but none of these two populations was significantly differentiated from Greeks and Czechs, who display an average value intermediate between those of Ethiopians and Omanis. Finally, the enzymatic activity measured for probe drug dextromethorphan (CYP2D6) stands out as the only one displaying no significant difference between any of the four populations tested.

**Fig. 3.**
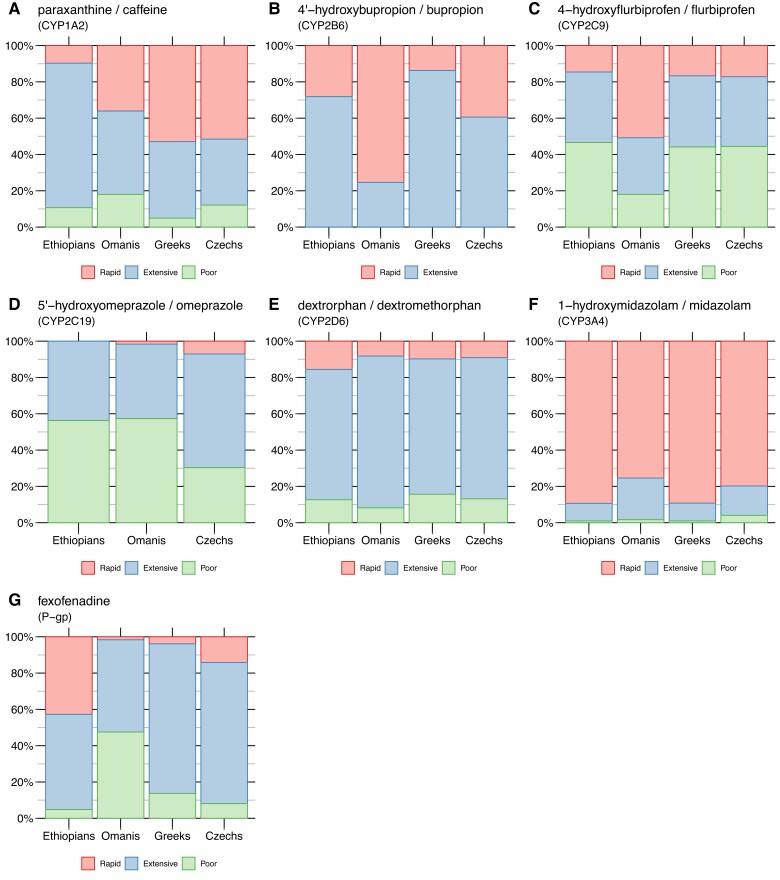
Proportions, for each population, of individuals in metabolizer categories of the seven compounds of the Geneva cocktail. (*A*) Caffeine. (*B*) Bupropion. (*C*) Flurbiprofen. (*D*) Omeprazole. (*E*) Dextromethorphan. (*F*) Midazolam. (*G*) Fexofenadine.

We then defined three discrete classes of enzymatic or transport activity (poor, extensive, or rapid metabolizers) for each of the seven compounds of the Geneva cocktail tested, using thresholds (shown in supplementary table S2, Supplementary Material online) based on previous knowledge acquired through inducer/inhibitor tests of the Geneva cocktail (Bosilkovska et al. 2014a, b). The proportions of individuals per population included in each of the three discrete phenotypic categories are reported in figure 3 and supplementary table S3, Supplementary Material online. Tests for equality of proportions between populations gave results broadly consistent with those obtained by comparing AUC distributions (supplementary table S4, Supplementary Material online), although differences were generally less pronounced (supplementary text, Supplementary Material online).

### Genome-wide Analyses of Associations

From the DNA extracted from saliva samples, and after quality controls (supplementary text, Supplementary Material online), we obtained the genotypes of 550,416 markers in 349 individuals (94 Ethiopians, 56 Omanis, 102 Greeks, and 97 Czechs), with an enriched Illumina CoreExome24 array. Using the same array and quality controls, we also obtained the genotypes of a sample of 119 Mandenka individuals from Senegal, whose DNA samples are studied in our laboratory since three decades (e.g., Tiercy et al. 1992, Poloni et al. 1995), and part of which is also included in the HGDP populations dataset (Bergström et al. 2020).

For each of the seven phenotypes measured in this study, a GWAS was performed with the GEMMA program (Zhou and Stephens, 2012), after the imputation of new markers with the Michigan Imputation Server using Minimac4 (Das et al. 2016), and log-transformed phenotypic data. Unless otherwise specified, all GWAS were performed considering age, sex, body mass index (BMI), and the five first principal components of a PCA (to account for population structure) as covariates (supplementary text, Supplementary Material online). The results of GWAS were mapped and analyzed using the Functional Mapping and Annotation (FUMA) web platform (Watanabe et al. 2017), including the MAGMA analysis (supplementary text, Supplementary Material online), so as to annotate candidate markers and prioritize candidate genes according to their genomic position (gene position mapping), their influence on the expression of given traits (eQTL mapping), and their influence on chromatin 3D interactions (3D chromatin mapping). The GWAS for phenotypes linked to the measured enzymatic activities encoded by the four genes of the CYP2 family, namely *CYP2B6*, *CYP2C9*, *CYP2C19,* and *CYP2D6*, led to significant signals.

**Fig. 4.**
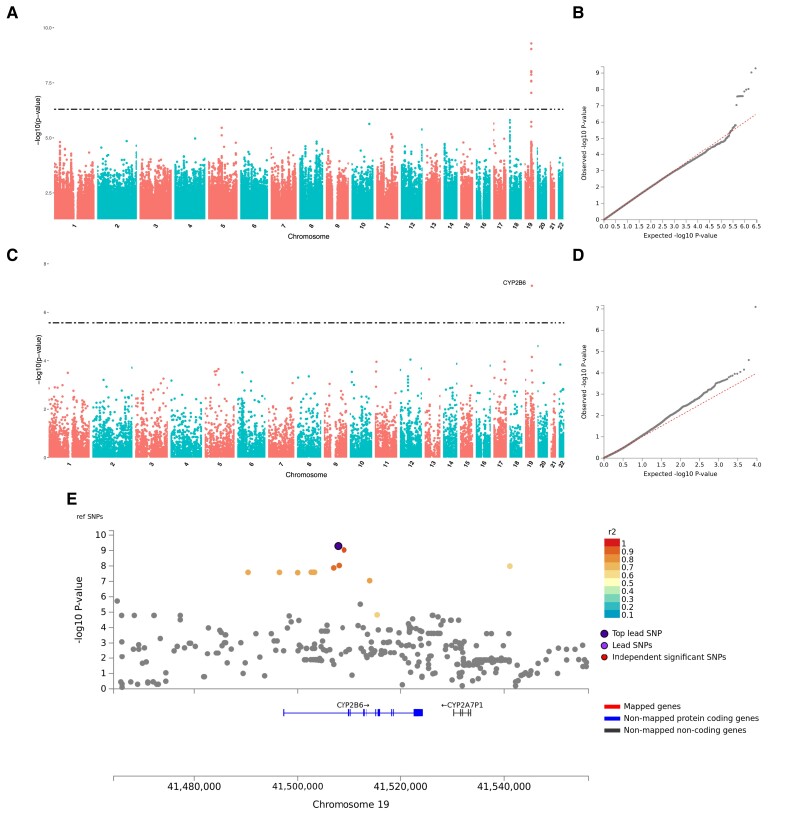
GWAS and FUMA gene-based GWAS for transformation rate of bupropion. (*A*) Manhattan plot of GWAS. (*B*) Q-Q plot of GWAS. (*C*) Manhattan plot of FUMA's gene-based GWAS. (*D*) Q-Q plot of FUMA's gene-based GWAS. (*E*) Regional plot of FUMA's identified genomic risk locus (GRL).

For the bupropion metabolism phenotype, GWAS results indicate several significant associations on chromosome 19, both with the classical GWAS (fig. 4*A*and*B*) and the FUMA gene-based GWAS (fig. 4*C*and*D*). FUMA identifies a single genomic risk locus (GRL) in this same area, spanning a 50 kb region encompassing *CYP2B6* and pseudogene *CYP2A7P1*, with the top lead SNP rs201788928 (which shares the same position with long indel rs59423078) located in the first intron of *CYP2B6* (fig. 4*E*, supplementary table S5, Supplementary Material online). *CYP2B6* is also significantly associated with the bupropion transformation phenotype in the MAGMA tissue expression analysis, which associates additional candidate markers with expression in the blood and spleen (supplementary fig. S1, Supplementary Material online). In mapping analyses, only that based on eQTL presents significant results corresponding to the same region evidenced as the GRL (supplementary table S6, Supplementary Material online).

**Fig. 5.**
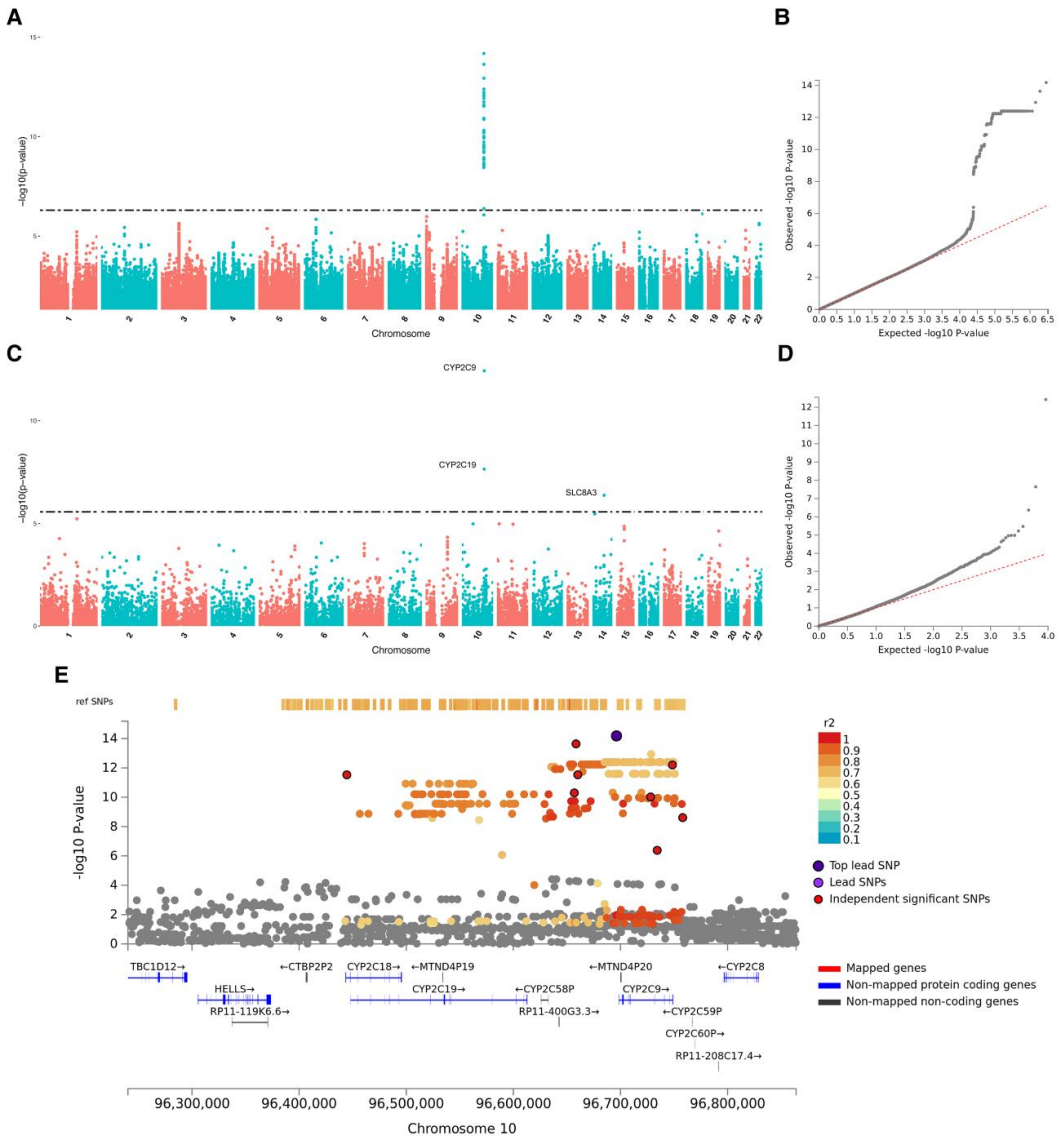
GWAS and FUMA gene-based GWAS for transformation rate of flurbiprofen. (*A*) Manhattan plot of GWAS. (*B*) Q-Q plot of GWAS. (*C*) Manhattan plot of FUMA's gene-based GWAS. (*D*) Q-Q plot of FUMA's gene-based GWAS. (*E*) Regional plot of FUMA's identified genomic risk locus (GRL).

In the GWAS of flurbiprofen metabolism, candidate markers are identified only on chromosome 10, in a region including gene *CYP2C9* (fig. 5*A*and*B*). On the other hand, FUMA gene-based GWAS points to an association with genes *CYP2C9* and *CYP2C19* on chromosome 10, as well as with *SLC8A3* on chromosome 14 (fig. 5*C*and*D*), but a single GRL is identified, which is characterized by the SNP lead rs34368405, an intergenic poly-A CNV located 2 kb upstream of *CYP2C9* (fig. 5*E*, supplementary table S7, Supplementary Material online). Finally, the mappings according to genomic position or influence on gene expression prioritize several different genes, all of them located in a 700 kb region around *CYP2C9* (Supplementary tables S8–S10, Supplementary Material online).

**Fig. 6.**
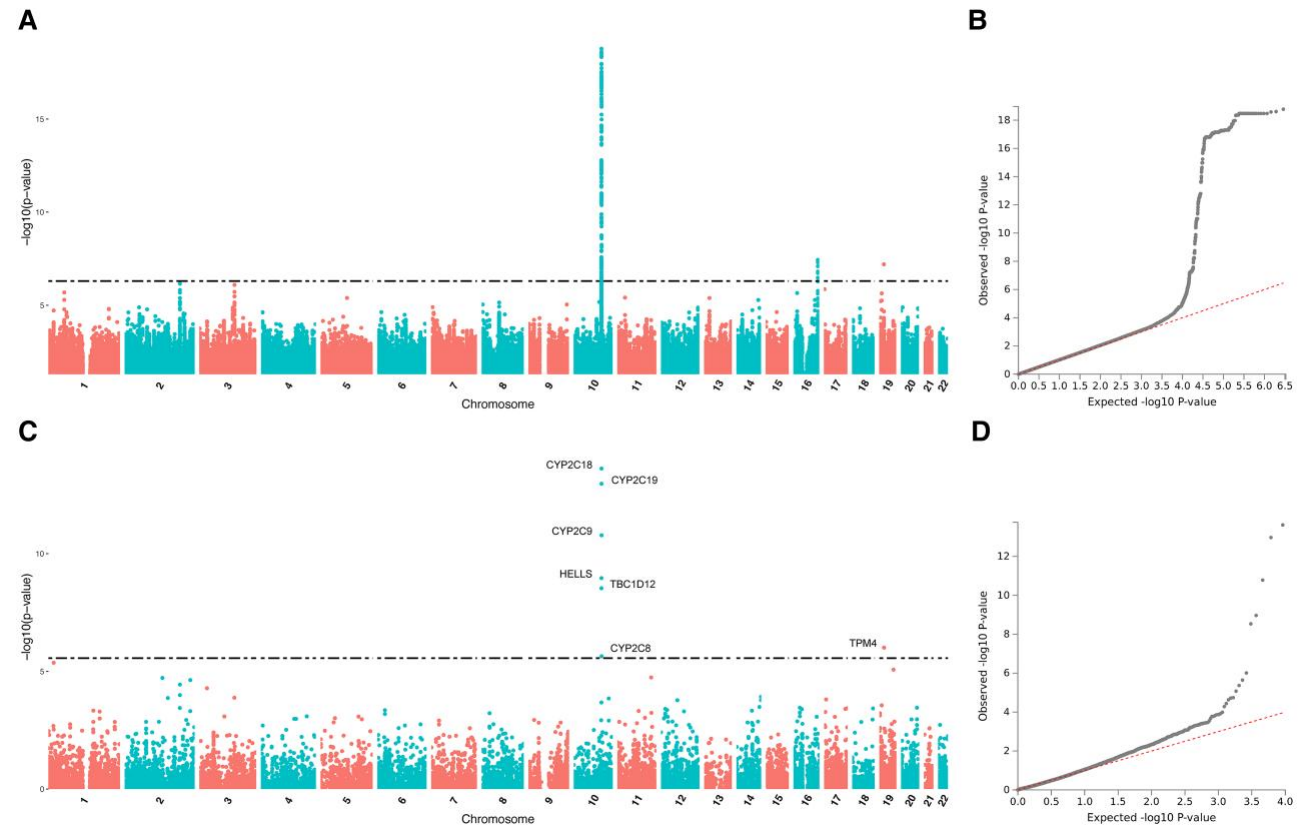
GWAS and FUMA gene-based GWAS for transformation rate of omeprazole. (*A*) Manhattan plot of GWAS. (*B*) Q-Q plot of GWAS. (*C*) Manhattan plot of FUMA's gene-based GWAS. (*D*) Q-Q plot of FUMA's gene-based GWAS.

Three areas indicating associations with the omeprazole transformation phenotype are identified by GWAS, on chromosomes 10, 16, and 19 (fig. 6*A*and*B*), whereas the FUMA gene-based GWAS indicates an association with genes around *CYP2C19* (on chromosome 10) and with *TPM4* on chromosome 19 (fig. 6*C*and*D*). The three GRLs identified by FUMA match the significant areas detected in the classical GWAS (supplementary fig. S2 and table S11, Supplementary Material online). The first GRL is supported by lead SNP rs12774450, located in an intergenic region before *CYP2C18*, 120 kb upstream of *CYP2C19* on chromosome 10 (supplementary fig. S2*A*, Supplementary Material online), the second GRL is supported by lead SNP rs9940513 on chromosome 16, also located in an intergenic stretch close to the pseudogene *COX6CP16* (supplementary fig. S2*B*, Supplementary Material online), and the third by lead SNP rs1415382173, located within intron 8 of gene *TPM4* on chromosome 19 (supplementary fig. S2*C*, Supplementary Material online). Among the three FUMA mapping analyses, only the eQTLq mapping leads to significant results, pointing to the same region evidenced by mapping for *CYP2C9* (supplementary table S12, Supplementary Material online).

**Fig. 7.**
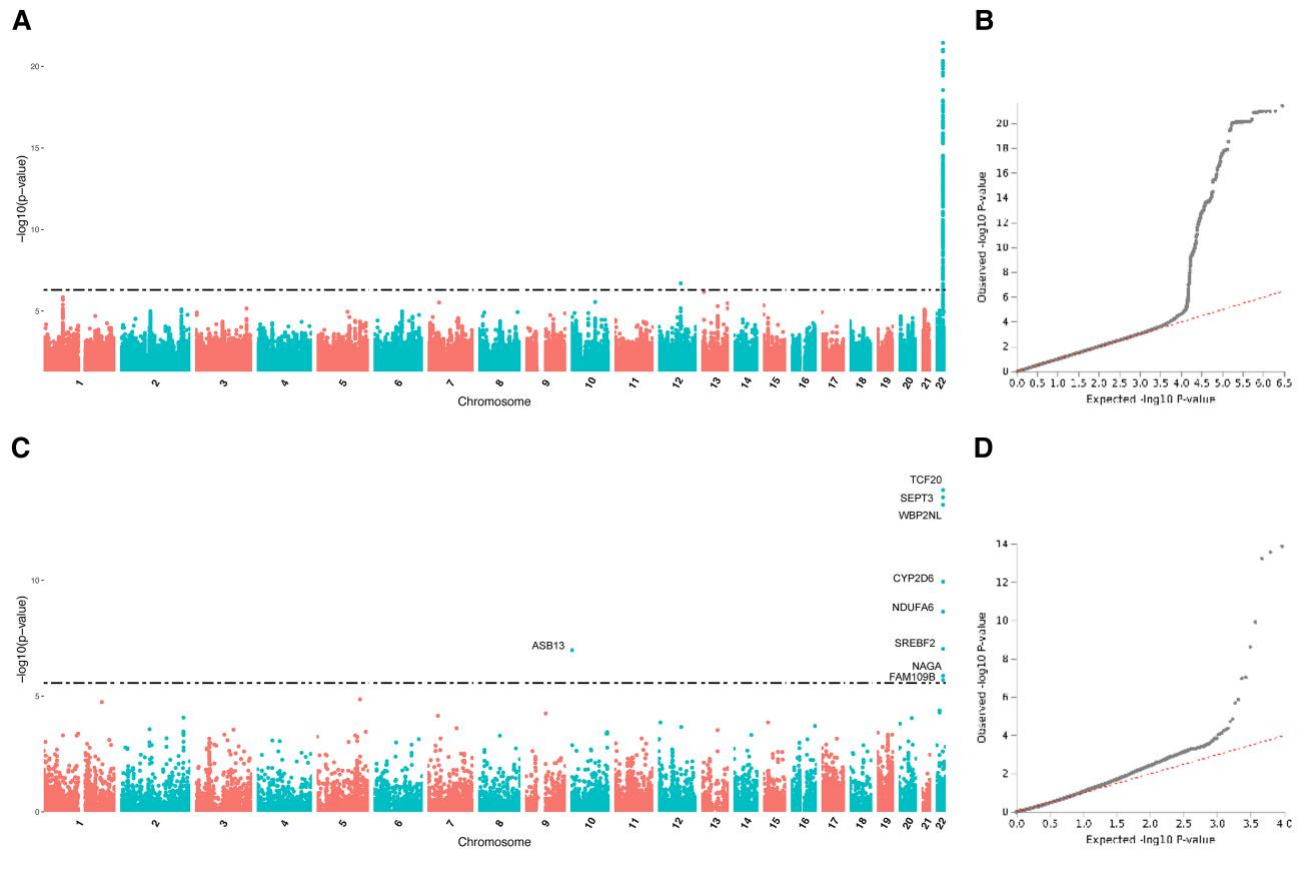
GWAS and FUMA gene-based GWAS for transformation rate of dextromethorphan. (*A*) Manhattan plot of GWAS. (*B*) Q-Q plot of GWAS. (*C*) Manhattan plot of FUMA's gene-based GWAS. (*D*) Q-Q plot of FUMA's gene-based GWAS.

For dextromethorphan metabolism, GWAS associates two regions with significant markers (fig. 7*A*and*B*), which are also identified by FUMA as GRLs (supplementary fig. S3 and table S13, Supplementary Material online). The first region is supported by rs201186564, an intronic indel of gene *NAV3* on chromosome 12 (supplementary fig. S3*A*, Supplementary Material online), whereas the second is located in the area of *CYP2D6* and is supported by three lead SNPs, rs4822079 (a regulatory region SNP between genes *SEPTIN3* and *WBP2NL*), rs13055841 (an intronic SNP in gene *SREBF2*), and rs28568508 (an intergenic variant lying between *CYP2D6* and the *CYP2D7P* pseudogene) as top lead (supplementary fig. S3*B*, Supplementary Material online). The FUMA gene-based GWAS also provides several genes associated with the phenotype in the *CYP2D6* area, plus *ASB13* on chromosome 10 (fig. 7*C*and*D*). Finally, the mapping analyses prioritize 24 genes by a combination of the three mapping methods, all located in a wide genomic area spanning almost 1.5 Mb around *CYP2D6* (supplementary tables S14–S16, Supplementary Material online).

Contrarily to the enzymatic activities encoded by genes of the CYP2 family (i.e., *CYP2B6*, *CYP2C9*, *CYP2C19,* and *CYP2D6*), the GWAS on phenotypes of caffeine and midazolam metabolism and fexofenadine transport, linked, respectively, to *CYP1A2* (supplementary fig. S4, Supplementary Material online), *CYP3A4* (supplementary fig. S5, Supplementary Material online), and *ABCB1* (supplementary fig. S6, Supplementary Material online) did not show any candidate marker. However, the MAGMA analysis of GWAS on *CYP1A2* activity provides a possible association with expression levels in the small intestine (supplementary fig. S7, Supplementary Material online). Similarly, a significant association between gene *CLYBL* (on chromosome 13) and the P-gp (fexofenadine transport) phenotype is output by the FUMA gene-based GWAS of *ABCB1* activity (Supplementary fig. S6*C*and*D*).

### Genomic Scans of Selection

Following the results of the phenotypic comparisons, we used three genomic approaches to identify genes potentially under selective pressures related to the metabolism of exogenous compounds.

**Fig. 8.**
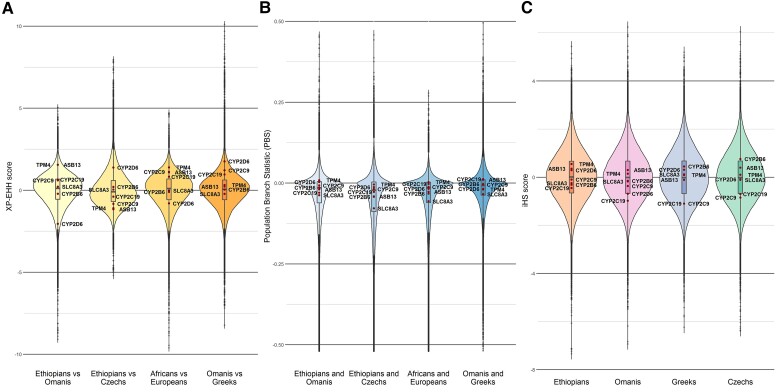
Violin plots of the results of the three genomic scans of selection. Outlier signals in the three scans (lying beyond the lower/upper distribution limits, i.e., beyond the first/third quartile ± 1.5 × interquartile range) are indicated by small gray dots located at the extremes of each distribution. The most extreme values correspond to candidate loci for selection. Values associated with genes identified by GWAS (*CYP2B6*, *CYP2C9*, *CYP2C19*, *CYP2D6*, *ASB13*, *SLC8A3,* and *TPM4*) are indicated by red dots and are all located around the center of each distribution. (*A*) XP-EHH scores. (*B*) PBS scores; negative scores beyond −0.52 are not shown. (*C*) iHS scores.

First, we compared the two most differentiated populations for each of the evaluated phenotypes, using the XP-EHH method (Sabeti et al. 2007). Four XP-EHH analyzes were thus carried out, comparing (1) Ethiopians with Omanis (ADD vs. MUS) for the flurbiprofen-, midazolam- and fexofenadine-related phenotypes, associated respectively with the measures of *CYP2C9*, *CYP3A4*, and *ABCB1* encoded activities (supplementary table S17, Supplementary Material online), (2) Ethiopians and Omanis with Greeks and Czechs (ADD + MUS vs. ALE + PRA) for the caffeine-related phenotype, associated with *CYP1A2* (supplementary table S18, Supplementary Material online), (3) Czechs with Ethiopians (PRA vs. ADD) for the omeprazole-related phenotype, associated with *CYP2C19* (supplementary table S19, Supplementary Material online), and (4) Omanis with Greeks (MUS vs. ALE) for the bupropion-related phenotype, associated with *CYP2B6* (supplementary table S20, Supplementary Material online). Note that this latter comparison was also used to reanalyze *CYP2C9*-associated phenotypic distributions (flurbiprofen transformation), due to their apparent skewness. In total, the four tests identify 46 candidate areas and 215 candidate genes (supplementary table S21, Supplementary Material online). None of the seven genes targeted by the Geneva cocktail was included in those candidate zones (fig. 8*A*).

As an alternative to XP-EHH, which is based on an analysis of variation in linkage disequilibrium, we also compared the same four populations with the population branch statistic (PBS) method (Yi et al. 2010), which is based on an analysis of *F*_ST_-measured changes in allele frequency. We applied the same sliding windows approach as in Fumagalli et al. (2015). Because this method detects signals of positive selection in a directional fashion, eight comparisons were performed (e.g., MUS vs. ADD, then ADD vs. MUS), always using the Mandenka genotype data (SEN) as the second reference population (supplementary text, Supplementary material online). Twelve candidate areas were outputted by the PBS analysis due to extreme change in *F*_ST_ in the target population, therefore, potentially indicating 12 regions that experienced population-specific positive selection (supplementary table S22 and fig. S8, Supplementary Material online). However, only two of these outputted candidate zones are in common with the XP-EHH results. One PBS peak points to a signal in the HLA cluster region on chromosome 6 (associated with HLA genes or with *BTNL2*, a gene also involved in the immune system) in the comparison of Ethiopians with Omanis, in that of Africans and Middle Easterners with Europeans, as well as in that of Omanis with Greeks (supplementary table S22, Supplementary Material online), and the other points to genes from the alcohol dehydrogenase (ADH) family on chromosome 4 in the comparison of Ethiopians with Czechs (supplementary tables S17 and S19, Supplementary Material online). Inversely, the strong signals associated either to the *GPHN* gene on chromosome 14 or the *DARC* gene region on chromosome 1 are not mirrored in the XP-EHH results. More importantly, none of the seven genes targeted by the Geneva cocktail was included in the PBS candidate zones (fig. 8*B*).

Finally, as a third approach, we used the iHS scores scan, designed to detect recent soft sweeps in specific populations, but potentially also signals of balancing selection (Voight et al. 2006). In comparison to the two methods above, iHS scores are computed for each of the four populations independently and do not require a reference population. We used the R package *rehh* (Gautier and Vitalis 2012) to compute iHS scores, and candidate regions were identified as in Vicente et al. (2019, supplementary text, Supplementary Material online). For each of the four populations tested, the strongest signal was provided by the *HLA* region on the short arm of chromosome 6 (supplementary table S23 and fig. S9, Supplementary Material online), thereby supporting the XP-EHH and PBS results. Besides the human MHC region, two other candidate regions (in a ∼400 Kb region near the centromere on the short arm of chromosome 1, and in a ∼1,400 Kb region between q12 and q13.3 on the long arm of chromosome 15, Supplementary tables S21 and S23 and fig. S9, Supplementary Material online) were found in common in the XP-EHH and iHS tests. On the other hand, those signals specific to the XP-EHH test (*ADH* genes cluster on chromosome 4, *DARC* on chromosome 1) are not mirrored in the iHS scans, and similar to the XP-EHH and PBS results, neither of the seven genes targeted by the Geneva cocktail is identified with the iHS approach (fig. 8*C*). Interestingly, however (and contrarily to the results of the two former approaches), a single match with the “evidence-based ADME genes” list of PharmaADME was found with iHS for the *NAT1* gene in the Omani population. This result is in line with previous studies reporting signals of directional selection in the sequence diversity of *NAT1* identified with classical tests of selective neutrality, such as Tajima's *D* test (Sabbagh et al. 2018, Vangenot et al. 2019).

## Discussion

This study presents phenotypic and genotypic data related to xenobiotic transformation and elimination, from four different populations, two of which come from a geographic area often underrepresented in studies of the pharmacogenetics or pharmacogenomics variability of humans (LLerena et al. 2014; Fricke-Galindo et al. 2016), namely Africa and the Middle East. In contrast to many previous studies that were mostly based on patient cohorts, our sample is exclusively composed of healthy individuals representing regional populations. The combination of two minimally invasive sampling methods that allow easy storage (at room temperature) of collected materials, that is DNA samples from saliva, and dried blood spots following consumption of a low-dose cocktail of drugs specifically developed for clinical phenotyping, provides an affordable streamlined approach for future studies in locations lacking extensive infrastructures. This protocol could, therefore, facilitate the future collection of data from populations that remain underrepresented in current ADME datasets, thereby increasing our comprehension of the function and evolution of those metabolic systems.

Our results demonstrate that there are significant population differences in metabolism rates. Indeed, of the seven phenotypes tested, six showed significant population differentiation, and only the phenotype associated with the activity of the *CYP2D6* gene revealed no significant differences among populations. For those six phenotypes showing some level of differentiation, we also found that the distribution of this variation does not follow some specific pattern, that is specific populations did not display consistently faster or slower average metabolic or transport activities for all phenotypes tested; on the contrary, each phenotype appears to have its own specificity with regard to the populations investigated.

### Differentiation Between Europe and the Middle East/Africa in Caffeine Demethylation Phenotype

The phenotype defined as caffeine demethylation into paraxanthine, the major metabolite of caffeine in humans, (Lelo et al. 1986; Gu et al. 1992), is attributed to a metabolic pathway that is primarily carried out by the enzyme encoded by *CYP1A2*. This is the only phenotype separating the four populations into two geographically distinct “continental” groups, with Europeans presenting higher average enzymatic activity than the other two populations. Among the latter, Ethiopians were found to have the slowest average caffeine metabolism rate, although this was not significantly slower than the average rate observed in Omanis (fig. 2*A*, supplementary table S1, Supplementary Material online). Coffee consumption has been previously shown to be an important factor affecting the expression of the *CYP1A2* gene (Tantcheva-Poór et al. 1999; Amin et al. 2012). *Coffea arabica*, the coffee plant species most commonly cultivated, is native to a region encompassing Ethiopia (Anthony et al. 2002; Scalabrin et al. 2020). Therefore, one could suppose that this dietary habit has influenced the phenotype, but another publication suggested that the higher observed molecular variability of Ethiopians for *CYP1A2* could also translate into a significant proportion of individuals lacking the corresponding enzymatic activity (Browning et al. 2010). A dietary influence would thus not be expected to go in the direction of reduced activity of the enzyme in Ethiopians, because strong coffee consumption has been shown to have an induction effect on *CYP1A2* (Ayalogu et al. 1995; Djordjevic et al. 2008). Indeed, our results show significantly less rapid metabolizers in Ethiopians, compared with the other populations, but more extensive metabolizers, and no individuals were found to have null activity. Consistently, our GWAS on this phenotype did not return any significant association, even with *CYP1A2*. Although some specific *CYP1A2* variants, whose allele frequency distributions differ between populations, have been identified as having an influence on this phenotype, it is generally held that the activity of *CYP1A2* is only slightly influenced by genetics, and rather largely by environmental factors (Faber et al. 2005; McGraw et al. 2018).

### No GWAS Association With the Oxidative Process of Midazolam

We found a few marked differences between populations for the phenotype based on the concentration of midazolam transformation to its major metabolite, hydroxymidazolam, associated with the enzymatic activity encoded by *CYP3A4*. Ethiopians are on average the fastest metabolizers and Omanis the slowest, but neither of these two populations shows any significant difference from the two European populations. GWAS on this phenotype failed to reveal any association with a genetic marker, which is consistent with previous studies that instead described the influence of other factors on the variability of CYP3A4 mediated metabolism, such as other inducing molecules, sex or inflammation (Zanger and Schwab, 2013). Therefore, it seems clear that genetic factors do not influence the enzymatic activity encoded by *CYP3A4* at the population level, which makes a possible local adaptation of this gene unlikely. On the other hand, it is possible that *CYP3A4* is under purifying selection, due to the enzyme being involved both in crucial functions of detoxification or excretion linked to the treatment of different (often toxic) substrates, as well as in various essential metabolism pathways such as the synthesis of steroid cholesterol and lipid compounds (Lee et al. 2001, 2002a; Elens et al. 2011). Based on the diversity captured by our array, *CYP3A4* indeed displays the lowest or among the lowest levels of nucleotide diversity compared with five other genes targeted by the Geneva cocktail (supplementary fig. S10, Supplementary Material online), consistent with the results of Zhou et al. (2017).

### No GWAS Association With the Transport of Fexofenadine

Similar to the metabolism of midazolam, no marker was associated with the transport of fexofenadine by GWAS. However, the case of *ABCB1* is somewhat different from that of cytochrome P450s, because this gene encodes the P-gp, a transmembrane transporter of the plasma barrier (Levran et al. 2008). The faster the P-gp works, the lower is the AUC of fexofenadine in the bloodstream. Our phenotypic comparison indicates that P-gp activity significantly differentiates the four populations from each other. Ethiopians display the highest fexofenadine average transport rate, and Omanis, the lowest. With regard to Ethiopians, our results could bring some support to those of the study by Kim (Kim, 2001), who found that African–American individuals exhibited a faster P-gp activity than American individuals of European origin, and linked this difference to genetic factors. We are aware, however, that such a comparison suffers from considering a close genetic similarity between Ethiopians and African–Americans, which is not supported by the current knowledge on genomic diversity in Africa (Pagani et al. 2012). For the Omanis, however, the lack of data from the Middle East does not allow comparison with the literature. As our study demonstrates that Omanis exhibit lower *ABCB1-*encoded enzymatic activity than Europeans, our results further stress the need to increase the genetic and genomic knowledge of this geographic area. While GWAS for fexofenadine concentration did not output any candidate marker, *CLYBL* was found to be associated with the phenotype in the context of the gene-based GWAS. The protein product of this gene takes part in vitamin B12 metabolism and has been jointly associated with *ABCB1* in a study of cancer cell drug resistance (Sherman-Baust et al. 2011).

### No Population Differences in Dextromethorphan Demethylation Phenotype but GWAS Association With two Candidate Regions

The demethylation of dextromethorphan, associated with the activity of *CYP2D6*, is the only phenotype examined in our study that did not reveal any difference between populations, neither when measured as a continuous phenotype (fig. 2) nor when classified into metabolizer categories (fig. 3). This result is surprising, given that *CYP2D6* is one of the most studied CYP genes, due to its considerable inter-individual variability and its importance as a pharmacogene (Zanger et al. 2004; Sistonen et al. 2007; LLerena et al. 2014; Gaedigk et al. 2017). However, most of those studies did not directly assess metabolic phenotypes, but rather relied on predictions from genetic data, that is genetic phenotypes. This raises concerns about the accuracy of genetically predicted phenotypic assignments, a problem probably linked to the structural complexity of the *CYP2D6* genomic region, which can include multiple polymorphic copies of the *CYP2D6* gene (Nofziger et al. 2020).

The GWAS of dextromethorphan demethylation identified two candidate zones. The first zone is located around *CYP2D6*, on chromosome 22, and presents a very strong signal that impacts a large genomic segment, which seems to confirm the reported importance of genetic factors responsible for inter-individual variability in enzymatic activity between individuals (Zanger et al. 2004; Magliocco et al. 2019). The second zone is supported by a marker located in the *NAV3* gene, on chromosome 12, which encodes a navigator neuron associated with some types of cancer and could be a good prognostic gene and therapeutic target (Carlsson et al. 2013). However, the link between *NAV3* and the enzymatic activity of *CYP2D6* is at present unclear. Investigating if the association signal in the *NAV3* genomic region is linked to the use of a contraceptive pill could be addressed in future studies. Indeed, a possible influence of the contraceptive pill on dextromethorphan metabolism, albeit small, is suggested by our sensitivity analyses (supplementary text and supplementary table S1, Supplementary Material online), and a GWAS including only female participants (considering the use of a contraceptive pill as a covariate, along with age, BMI and the five first principal components of the PCA) did only reproduce the strong association signal of the phenotype with the genomic region of *CYP2D6*, on chromosome 22 (supplementary fig. S11, Supplementary Material online). In this additional GWAS, the association level of the region encompassing *NAV3*, on chromosome 12, fails to reach the significance threshold, although such a loss of signal might merely result from a loss of power due to the considerable reduction in sample size (∼44%), when analyzing only women.

The gene-based GWAS outputs *ASB13*, located on chromosome 10, as a candidate gene in addition to *CYP2D6*. *ASB13* encodes a protein containing an ankyrin repeat motif and a SOCS box domain that targets proteins in order to proceed to their proteasomal-mediated degradation (Anasa et al. 2018). At present, there is no known link between metabolism by CYP enzymes and the ASB protein family, but this might be a field to explore in light of the involvement of ASBs in the pathway of ubiquitylation and degradation of proteins. Interestingly, *CYP2D6* also functions to metabolize many important endogenous substrates like dopamine from hydroxytryptamines, neurosteroids, and both m-tyramine and p-tyramine (Wang et al. 2009, 2014). It has been hypothesized that differences in *CYP2D6* allelic frequencies between some African populations could reflect selection for the metabolism of alkaloids to provide a source of nutrients under scarce food resource conditions (Ingelman-Sundberg, 2005). This hypothesis is, however, based on the assumption that allelic frequency differences do lead to differences in enzymatic activity between populations, which were not demonstrated in our study. Instead, given the many endogenous functions that *CYP2D6* fulfills, the lack of phenotypic population structure found in our study could be due to a selective process acting on standing *CYP2D6* genetic variation to maintain a similar *CYP2D6* encoded function in all populations. Other studies have additionally reported that the *POR* gene also contributes to the variability of *CYP2D6* activity (Sandee et al. 2010), but no signal from this gene was detected in our GWAS or prioritized by FUMA results.

### Hydroxylation of Bupropion is Directly Associated With *CYP2B6* by GWAS and is on Average Slower Among Greeks and Faster Among Omanis

The only phenotype associated with a cytochrome P450 gene, for which a difference is observed between all populations of our study, corresponds to the rate of hydroxylation of bupropion into hydroxybupropion, primarily undertaken by the hepatic enzyme encoded by *CYP2B6* (Hesse et al. 2000). The population with the slowest average enzymatic activity in our study is the Greek population (fig. 2*B*, supplementary table S1, Supplementary Material online). This observation is somewhat at odds with previous investigations reporting higher frequencies of low-activity *CYP2B6* alleles in African populations (Klein et al. 2005; Zanger and Klein, 2013), although Ethiopians were also found to display an average slower metabolism than Czechs and Omanis. However, although the four populations were found to be phenotypically differentiated, no individual in our cohort was classified as a poor metabolizer. The highest average enzymatic activity was determined for the Omani population. We could not corroborate this finding based on the literature as there is (to the best of our knowledge) an apparent scarcity of ADME studies on Arabian populations, either from a phenotypic or a genotypic perspective. At present, according to a review available, Arab populations genotyped for *CYP2B6* do not show any variation in frequency distributions compared with European populations (Ossaily and Zgheib, 2014). Based on our GWAS results, *CYP2B6* is the only gene associated with burpropion hydroxylation activity, which supports the influence of *CYP2B6* genetics on individual variability of this phenotype (Naidoo et al. 2014).

### GWAS Association of Flurbiprofen Hydroxylation Phenotype With *CYP2C9* and Possible Links With Calcium-dependent Enzymes

On average, the Omanis are faster than the other three populations in the hydroxylation of flurbiprofen into hydroxyflurbiprofen, a metabolic function carried out by the enzyme encoded by *CYP2C9*. GWAS only identified a single genomic region displaying a very strong association with the phenotype, which is located around *CYP2C9*, thus validating the influence of the gene's variation on the phenotype. This result is interesting because, although differences in allelic frequency between populations have been reported (Lee et al. 2002b; Sistonen et al. 2009), the link between genetics and phenotypic variation for *CYP2C9* is not conclusive (Céspedes-Garro et al. 2015; Wang et al. 2020), and requires confirmation with measured metabolic phenotypes. In the gene-based GWAS output from FUMA, both *CYP2C9* and *CYP2C19* are indicated, as well as *SLC8A3* (Solute Carrier Family 8 Member A3). *SLC8A3* encodes the sodium/calcium exchanger 3 protein (also known as NCX3), a plasma-membrane protein that mediates the exchange of sodium/calcium ions across the cell membrane (and is, therefore, involved in calcium-dependent cellular processes). However, we could not identify any apparent link between this gene and the activity of *CYP2C9*. It is nevertheless interesting to note that other sodium/calcium exchanger genes have been previously categorized as ADME genes (pharmaadme.org).

### Faster Oxidation of Omeprazole Among Czechs and GWAS Association With Region Displaying Large-scale Structural Variation

The enzymatic activity of CYP2C19 is responsible for the oxidation of omeprazole and was found slightly, but significantly, faster in Czechs than in the other tested populations. Compared with phenotypic predictions based on markers leading to poor or rapid metabolism (Fricke-Galindo et al. 2016), our results show an over-representation of poor metabolizers and an under-representation of rapid metabolizers, relative to expected proportions from the literature (Zhou and Stephens, 2012; Fricke-Galindo et al. 2016). This is particularly the case for Ethiopians and Omanis. However, once again, confrontation of these results with former knowledge is hampered by the scarcity of comparable data on African and Middle Eastern populations.

Three distinct genomic regions are identified as associated with the phenotype in GWAS (fig. 6). Despite that the sensitivity analyses results are suggestive (at best) of an influence of alcohol consumption on omeprazole metabolism (supplementary text and supplementary table S1, Supplementary Material online), the same three association signals are reproduced in a GWAS that also includes alcohol consumption among covariates (supplementary fig. S12, Supplementary Material online). The first one is the area around *CYP2C19*, which confirms that the examined phenotype is at least partly determined by distinct genetic variants in that gene (Fuhr et al. 2007). The second link highlighted by GWAS with the *TPM4* region on chromosome 19 is surprising, as this particular gene encodes an enzyme of the actin-binding tropomyosin family, involved in the function of actin filaments in cells. Likewise, the third associated region is located near the *COX6CP16* pseudogene, within a region on chromosome 16 previously identified as demonstrating large-scale copy-number variations across human genomes (Iafrate et al. 2004), but its association with the phenotype remains at present elusive.

### Influence of the Environment

Our GWAS of seven representative drug-metabolizing phenotypes revealed three different patterns. The first involves caffeine, midazolam, and fexofenadine, three compounds for which phenotypic differences between populations were evidenced, but no GWAS signal was found. These phenotypes are attributed to the enzymatic activities associated with *CYP1A2*, *CYP3A4,* and *ABCB1* genes, respectively. For the two cytochrome P450 genes of this group, the absence of a GWAS signal is not surprising because the literature ascribes differences in activity (and/or expression) primarily to environmental and physiological factors (such as age and sex), rather than to specific genetic factors (Faber et al. 2005; Zanger and Klein, 2013; McGraw et al. 2018). For *ABCB1*, the picture is less clear, because several studies have identified variants linked to the prevalence of certain cancers and the outcome of treatments, and some of those variants could be under selection (Wang et al. 2007; Sychev et al. 2018; Han et al. 2020). However, the lack of signal in our GWAS indicates that the activity of these three genes may not be primarily determined by genetic factors, meaning that the observed phenotypic differences between populations are likely to be due to environmental factors such as the consumption of inhibitory or inducing compounds through the diet. For example, induction by heavy caffeine consumption is known for *CYP1A2*, as is the induction of *CYP3A4* by certain botanical compounds of herbs (Raucy, 2003). Thus, the observed differentiated metabolic rates among populations of phenotypes associated with *CYP1A2*, *CYP3A4*, and *ABCB1* possibly reflect phenotypic plasticity. On the other hand, it is also possible that the enzymatic activity is influenced by multiple genes, but GWAS is an approach known to lack the power to detect complex multigenic associations (Eichler et al. 2010; Gibson, 2012).

Dextromethorphan represents the second pattern, characterized by the absence of phenotypic differences between populations, but displaying a GWAS signal including *CYP2D6*, thereby supporting current knowledge about the influence of *CYP2D6* genetic variants on the encoded enzymatic activity. Moreover, our FUMA results suggest the involvement of additional mechanisms in the resulting phenotype, such as expression modulation that may be attributed to a broad candidate genomic region identified by mapping, eQTL, and chromatin interaction analyses. Although local adaptive selective pressures could act on any of the candidate genes in this region, the observed lack of phenotypic differences among populations disfavors this hypothesis. Moreover, the large variance of phenotypes within populations that we found in this study, combined with the high genetic diversity known for this gene (Ingelman-Sundberg, 2005), is more likely to be compatible with the action of some form of balancing selection maintaining a necessary function, characterized by the capacity to metabolize a broad spectrum of different molecules, irrespective of environmental influences. Consistently, CYP2D6 is known both for its involvement in the metabolism of about 20% of drugs and its low inducibility by xenobiotic exposure (Benedetti, 2000).

Finally, the third pattern was evidenced for *CYP2B6*, *CYP2C9*, and *CYP2C19*, for which we found both phenotypic differentiation between populations and significant GWAS signals. This pattern indicates that genetic factors, indeed, influence the enzymatic activity of the products of those genes, and raises the possibility that the observed phenotypic differentiation between populations is due to differentiated allele frequency distributions. Thus, this third pattern is the most consistent with the existence of local adaptation in different populations. It could also be due, however, to local environmental (non-genetic) factors shaping inter-population variability, with genetic factors being only responsible for inter-individual variability, thereby in line with the lack of selection signals found in our XP-EHH, PBS, and iHS scans (fig. 8). In this respect, previous studies have shown that the genetic structure of populations for both *CYP2C19* (and *CYP2D6* too) is consistent with the main demographic model of human expansion over the globe, whereas this is not the case for *CYP2C9* (Sistonen et al. 2007, 2009). Consistently, in many populations analyzed worldwide on the basis of annotated star alleles, few individuals are predicted to exhibit markedly decreased CYP2C9 function, suggesting that purifying selection may act on the gene to prevent the accumulation of deleterious variation (Céspedes-Garro et al. 2015; Zhou et al. 2017).

Despite that numerous candidate genes were output by our XP-EHH tests (supplementary table S21, Supplementary Material online), only a few ADME genes were included among those, namely *MGST2*, *PON1*, *PON3*, *ADH,* and *CYP21A2*. The signal of positive selection on *MGST2* in Ethiopians and Omanis (supplementary table S18, Supplementary Material online), a gene involved in the detoxification of xenobiotics (Hayes et al. 2005; Uno et al. 2013), was already found in high altitude Asian and American populations (Foll et al. 2014). The two *PON* genes, associated with a significant signal in Omanis (supplementary table S17, Supplementary Material online), are members of the paraoxonase family of enzymes involved in the hydrolysis of thiolactones and xenobiotics (van Himbergen, 2006). The natural substrates of PON enzymes appear to be lactones (Khersonsky and Tawfik, 2005), which are found in many foods and influence their taste and smell (Berger, 2007). Moreover, these enzymes are also involved in the arachidonic acid pathway, similar to CYP3A4 (Adams et al. 1996). As expected from previous knowledge on African populations (Johnson and Voight, 2018), genes from the ADH family showed signals of positive selection in Ethiopians (Supplementary tables S17 and S19, Supplementary Material online). Finally, the *CYP21A2* gene, which was output by XP-EHH as selected in Omanis (supplementary table S20, Supplementary Material online), is well known for its role in cortisol and aldosterone synthesis in the adrenal glands, and deficiency in this gene is the major factor of congenital adrenal hyperplasia (Soardi et al. 2008). This signal in *CYP21A2* was not detected in the PBS scans, which in turn produced a signal located some 400 kb away, possibly related to *HLA* genes or the immunoregulator gene *BTNL2*, thereby suggesting, both in Ethiopians and Omanis, possible adaptions to specific pathogenic environments (Sanchez-Mazas 2020). Such potential signals of adaptation are sustained by the iHS scan results, which output the *HLA* region on the short arm of chromosome 6 in all four tested populations. Besides HLA, however, we found little overlapping between the results of the three scan of selection approaches, which indicates that candidate signals of XP-EHH and iHS, located in regions of unusually extended homozygosity (and hence, strong linkage disequilibrium), do not correspond in our dataset with regions of unusually large allele frequency change that would be detected as PBS signals. We are aware that the detection threshold chosen in the PBS scans is very stringent (99.99th percentile, supplementary text, Supplementary Material online), but lowering this threshold would inflate disproportionally the false discovery rate (supplementary fig. S8, Supplementary Material online). Besides the region on chromosome 6 discussed here above, which was also detected as selected in Omanis by XP-EHH (supplementary tables S17 and S20, Supplementary Material online), a single additional concordant output of the two approaches is a PBS signal found for an ADH gene (*ADH7*, supplementary table S22 and fig. S8, Supplementary Material online, Supplementary Material online), whereas the XP-EHH signals in *MGST2* and *PON* genes were not detected in PBS analyses. Independent replication of these results is needed, especially so for the region including the *PON* genes, since (to the best of our knowledge) it has never been identified before.

Note that methods such as XP-EHH and PBS are able to detect signals of strong directional selection on variants that are close to fixation, whereas iHS has the potential to detect softer selective sweeps, and also balancing selection (Voight et al. 2006). The action of this latter mechanism on the evolution of the diversity of some ADME genes is probably relevant to consider, notably for those genes coding for enzymes with an important number of different exogenous substrates. However, besides the candidate signal in the *NAT1* gene region in Omanis, no other ADME gene was identified by our iHS scans.

More generally, the link of the candidate regions identified by XP-EHH, PBS, or iHS with a possible adaptation to the chemical environment is not obvious and requires further investigation. While endogenous molecules that are substrates of the enzymes and the transporter targeted by the Geneva cocktail are fairly well known, information on natural exogenous substrates is limited. Undoubtedly, such knowledge would be very useful in order to establish new or clarify existing hypotheses regarding the selective pressures acting on these xenobiotic metabolizing enzymes or transport systems. For example, tyramines, which are one of the natural substrates of *CYP2D6*, are contained in many fermented foods and are used by the organism for dopamine biosynthesis, although they can also have a nefarious effect because high consumption of this type of molecules can cause hypertensive crises (Wang et al. 2014). If the natural function of *CYP2D6* is important to avoid such toxic effects, this could explain why we do not observe a phenotypic difference between populations. Indeed, for *CYP2D6*, previous studies failed to reveal any local selection phenomena, and differences in allele frequencies between populations are consistent with human demographic history (Sistonen et al. 2007). Note that the previous hypothesis that *CYP2D6* copy number increase (i.e., the multiplication of active alleles) in East African populations reflects local adaptation to a diet rich in toxic alkaloids (Ingelman-Sundberg, 2005) is not supported by the lack of phenotypic differentiation evidenced in our study. Even though a small tendency toward an increased prevalence of rapid metabolizers is observed among Ethiopians (fig. 3), this difference is not significant relative to the three other populations studied (supplementary table S4, Supplementary Material online).

The CYP genes are involved in the metabolism of different classes of fatty acids, including the synthesis of cholesterol, steroids, and other lipids (Miners and Birkett, 1998; Zhou et al. 2009; Magliocco et al. 2019). It is thus possible that observed phenotypic differences between populations (except apparently for *CYP2D6*), and their evidenced association with genetic variants, were shaped by local adaptations to diets with different compositions in fat. As proposed above, while *CYP2C9* may be subject to directional selection (either adaptive or purifying), this does not seem to be the case for *CYP2C19* (Sistonen et al. 2009). Other genes like *CYP1A2* are more likely to be subject to purifying selection, while positive selection acting on a few specific *CYP3A4* and *ABCB1* variants in some African populations has also been evidenced (Wang et al. 2007). Furthermore, many natural inhibitors or inducers of cytochrome P450 genes are known (Raucy, 2003; Zhou et al. 2009; He et al. 2015; Wang et al. 2020). Consequently, links between population genetics and phenotypes, namely population differences in haplotypic and phenotypic frequencies can be even more complex when the influence of the environment, which may be hard to identify, is considered. Indeed, differences in the chemical environments in which populations have lived for many generations can be expected among the places sampled in this study, which are located along a south-to-north latitudinal transect (fig. 1*A*). However, our results do not support the hypothesis that environmental variables correlating with latitudinal distance influenced the diversity of phenotypes, because in such case, the greatest differences would be expected between the two extremes of the transect, namely between Ethiopians and Czechs. The only phenotype for which such a tendency could be suggested is omeprazole transformation, in which *CYP2C19* (along with other potential genetic factors) is involved. However, as no signal of local (directional) selection in the ADME genes targeted by the Geneva cocktail was indicated by our XP-EHH and PBS tests on the phenotypically most differentiated populations, and not either by the population-specific iHS test (fig. 8), the notion that the diversity of these genes has been shaped by a strong adaptive process (at least strong positive selection) seems unlikely.

We are aware that due to the limited sample size, the lack of power in GWAS might be a possible explanation for the lack of association of caffeine, midazolam, and fexofenadine metabolisms with the genetic diversity of *CYP1A2*, *CYP3A4,* and *ABCB1*, respectively. In such case, however, our study indicates that the association of bupropion, flurbiprofen, omeprazole, and dextromethorphan metabolisms with *CYP2B6*, *CYP2C9*, *CYP2C19,* and *CYP2D6*, respectively, are rather strong, whereas the former ones would be expected to contribute only slightly, at best, to the corresponding phenotypes. We also acknowledge that genomic scans of selection, such as those performed in this study, suffer from several limitations, a major one being that neutral demographic processes such as bottlenecks or admixture can generate false positives signals, that is signals that can be confused with those resulting from adaptive selection (Harris et al. 2018; Apata and Pfeifer, 2020; Cuadros-Espinoza et al. 2022). We note however that, with respect to the ADME genes targeted by the Geneva cocktail or to ADME genes in general, our study rather reveals a lack of such signals (i.e., a potential excess of false negatives), suggesting a lack of power to detect soft sweeps or more complex selective mechanisms such as epistasis (Pritchard et al. 2010).

## Conclusion

Although the precise mechanisms that influence the evolution of ADME genes, more specifically of the seven genes targeted by the Geneva cocktail, are not yet fully identified, our study shows that they are likely to be fairly complex. The population approach adopted in this study, which analyzed both phenotypic and genotypic data, provided some essential insights into these systems. First, we demonstrated the existence of differences in average enzymatic activities between populations for six of the seven phenotypes examined. Second, we evidenced either a lack of any apparent genetic-phenotypic association (i.e., *CYP1A2* and caffeine, *CYP3A4* and midazolam, and *ABCB1* and fexofenadine, respectively), or the expected single-gene association (i.e., bupropion and *CYP2B6*, and flurbiprofen and *CYP2C9*, respectively), or more complex associations additional to those involving the primary gene (dextromethorphan and omeprazole metabolism). Our results, therefore, highlight different patterns of influence of genetic and/or environmental factors on each of the ADME systems examined.

## Materials and Methods

Sampling sites in four countries, that is Ethiopia, Oman, Greece, and the Czech Republic (fig. 1), were chosen because of their geographical location along a latitudinal transect and the expansion routes of modern humans out of Africa (Ramachandran et al. 2005; Li et al. 2008). In total, 367 individuals were sampled, 365 of which were successfully phenotyped (supplementary table S24, Supplementary Material online), and 349 among the latter were also successfully genotyped (table 1). The complete sampling and study protocol (deposit ID NCT02789527, at https://clinicaltrials.gov/) was approved by the competent ethics commissions and is described in the supplementary text, Supplementary material online.

**Table 1:** Summary of Information Provided by the 349 Participants to the Study Whose Sample was Successfully Phenotyped and Genotyped

	Ethiopia (ADD)		Oman (MUS)		Greece (ALE)		Czech Republic (PRA)		Total	
Sample size	94		56		102		97		349	
	**counts**	**proportion**	**counts**	**proportion**	**counts**	**proportion**	**counts**	**proportion**	**counts**	**proportion**
Female sample size	27	0.29	41	0.73	63	0.62	67	0.69	198	0.57
Male sample size	67	0.71	15	0.27	39	0.38	30	0.31	151	0.43
	**mean**	**s.d.**	**mean**	**s.d.**	**mean**	**s.d.**	**mean**	**s.d.**	**mean**	**s.d.**
Age (years)	21.2	2.3	26.9	6.4	21.9	4.1	25.0	4.8	23.4	4.9
Age (years), female	20.9	1.9	25.0	5.0	22.0	3.8	24.3	4.1	23.3	4.2
Age (years), male	21.3	2.4	31.9	7.3	21.7	4.7	26.4	6.1	23.5	5.7
Height (cm)^a^	170.4	8.7	160.5	9.5	171.7	8.9	171.9	9.5	169.6	9.9
Height (cm), female^a^	162.1	7.3	157.7	9.2	167.0	6.1	167.4	6.6	164.6	8.1
Height (cm), male	173.8	6.7	168.1	5.7	179.3	7.2	181.8	7.4	176.2	8.0
Weight (kg)	60.8	9.3	60.6	13.3	68.5	14.7	66.8	14.0	64.7	13.4
Weight (kg), female	54.5	7.7	55.4	9.3	62.6	9.7	61.8	10.6	59.7	10.2
Weight (kg), male	63.4	8.6	74.8	12.5	78.1	16.5	77.8	14.7	71.2	14.4
	**counts**	**proportion**	**counts**	**proportion**	**counts**	**proportion**	**counts**	**proportion**	**counts**	**proportion**
Smoking	1	0.01	0	0	17	0.17	24	0.25	42	0.12
Smoking daily	0	0	0	0	14	0.14	12	0.12	26	0.07
Smoking weekly	0	0	0	0	2	0.02	2	0.02	4	0.01
Smoking occasionally	1	0.01	0	0	1	0.01	10	0.10	12	0.03
Smoking, female	0	0	0	0	12	0.19	16	0.24	28	0.14
Smoking daily, female	0	0	0	0	11	0.17	6	0.09	17	0.09
Smoking weekly, female	0	0	0	0	1	0.02	1	0.01	2	0.01
Smoking occasionally, female	0	0	0	0	0	0	9	0.13	9	0.05
Smoking, male	1	0.01	0	0	5	0.13	8	0.27	14	0.09
Smoking daily, male	0	0	0	0	3	0.08	6	0.20	9	0.06
Smoking weekly, Male	0	0	0	0	1	0.03	1	0.03	2	0.01
Smoking occasionally, male	1	0.01	0	0	1	0.03	1	0.03	3	0.02
Drinking alcohol	24	0.26	0	0	84	0.82	94	0.97	202	0.58
Drinking alcohol daily	1	0.01	0	0	0	0	4	0.04	5	0.01
Drinking alcohol weekly	0	0	0	0	62	0.61	6	0.06	68	0.19
Drinking alcohol occasionally	23	0.24	0	0	22	0.22	84	0.87	129	0.37
Drinking alcohol, female	1	0.04	0	0	52	0.83	65	0.97	118	0.60
Drinking alcohol daily, female	0	0	0	0	0	0	0	0	0	0
Drinking alcohol weekly, female	0	0	0	0	35	0.56	5	0.07	40	0.20
Drinking alcohol occasionally, female	1	0.04	0	0	17	0.27	60	0.90	78	0.39
Drinking alcohol, male	23	0.34	0	0	32	0.82	29	0.97	84	0.56
Drinking alcohol daily, male	1	0.01	0	0	0	0	4	0.13	5	0.03
Drinking alcohol weekly, male	0	0	0	0	27	0.69	1	0.03	28	0.19
Drinking alcohol occasionally, male	22	0.33	0	0	5	0.13	24	0.80	51	0.34
Chewing khat	1	0.01	0	0	0	0	0	0	1	< 0.01
Chewing khat, male	1	0.01	0	0	0	0	0	0	1	0.01
Female sample size on contraceptive pill	0	0	0	0	7	0.11	29	0.43	36	0.18
History of antihistamine treatment^b^	0	0	0	0	0	0	7	0.07	7	0.02
History of antihistamine treatment, female	0	0	0	0	0	0	6	0.09	6	0.03
History of antihistamine treatment, male	0	0	0	0	0	0	1	0.03	1	0.01
History of thyroid medication/pathology^b^	0	0	0	0	1	0.01	3	0.03	4	0.01
History of thyroid medication/pathology, female	0	0	0	0	1	0.02	3	0.04	4	0.02
History of thyroid medication/pathology, male	0	0	0	0	0	0	0	0	0	0
Adverse events^c^	2	0.02	1	0.02	3	0.03	0	0	6	0.02
Adverse events, female	1	0.04	1	0.02	2	0.03	0	0	4	0.02
Adverse events, male	1	0.01	0	0	1	0.03	0	0	2	0.01

a One MUS female missing value.

b Individuals on actual antihistamine or thyroid medication were asked to stop their treatment 72 h before being sampled.

c An adverse event is defined as an untoward medical occurrence in a participant administered with a pharmaceutical product and which does not necessarily have a causal relationship with the study procedure. An adverse event can, therefore, be any unfavorable and unintended sign, symptom, or disease temporally associated with the use of a medicinal (investigational) product, whether or not related to the medicinal (investigational) product. Adverse events, described in Rollason et al. (2020), were all categorized as mild to moderate, non-serious, and resolved spontaneously.

### Phenotypes

Phenotypes were computed from concentrations of substrate drugs and their corresponding metabolites in blood samples collected at +2, + 3, and +6 h after ingestion of the seven probe-drug compounds’ Geneva cocktail. These phenotypes are AUC ratios and represent the rate of metabolization of each molecule in each individual. In addition, on the basis of his/her AUC ratio, each individual was assigned to one of three discrete classes (poor, extensive, rapid) of enzymatic or transporter activity. A full description of the method used for the computation of phenotypes is presented in supplementary text, Supplementary Material online.

### Population Comparisons of Phenotypic Distributions

For each of the seven phenotypes, populations were compared with Kruskal–Wallis and Wilcoxon two-by-two tests to highlight possible differences in AUC distributions, whereas proportion tests were applied to the discrete phenotypes datasets. Extensive analysis of potential confounders was performed by sensitivity analyses that compared subsets of populations, such as for example only women, only individuals not smoking, etc. (supplementary text, supplementary table S1 and supplementary figs. 11–19, Supplementary Material online).

### Genome-wide Genotypes

DNA extracted from collected saliva samples was processed with an Illumina CoreExome24 array including 555,356 built-in markers that we further enriched with 4,000 custom markers (supplementary table S25, Supplementary Material online). A full description of DNA preparation and processing, including all undertaken quality control filtering steps, is provided in the supplementary text, , Supplementary Material online. The resulting final dataset, referred to as the ADME dataset, is composed of 349 genotyped DNA samples for 550,416 markers. The same markers were also successfully genotyped in an in-house collection of DNA samples from 119 Senegalese Mandenka, referred to as the SEN dataset (supplementary text, Supplementary Material online). To check the quality of the data and control for population structure, we ran principal components analyses (PCA) and ADMIXTURE (Alexander et al. 2009) on our dataset of five populations, as well as an additional PCA with 19 samples of populations from the 1,000 Genomes (1KG) project dataset (1000 Genomes Project Consortium, 2015) as a reference (fig. 1*B*, supplementary text, and Supplementary figs. 20 and 21, Supplementary Material online).

### GWAS

The data from the 349 samples that were successfully phenotyped and genotyped (94 Ethiopians, 56 Omanis, 102 Greeks, and 97 Czechs) were used as input for genome-wide association analyses. Prior to GWAS, new markers were imputed from our genotyping data with the Michigan Imputation Server (MIS), using Minimac4 (Das et al. 2016), and a minor allele frequency (MAF) *>* 0.05, leading to a total of 5,773,125 markers (supplementary text, Supplementary Material online). We used the mixed linear model implemented in the GEMMA program (Zhou and Stephens, 2012) to perform seven GWAS, that is one for each of the seven compounds included in the Geneva cocktail. Covariates considered were age, sex, and BMI, as well as the five first principal components of the PCA (supplementary fig. S20, Supplementary Material online) to reduce bias from population structure. In order to increase power, the phenotypic data were log-transformed. Markers that showed an association with an associated *P-value* < 5 × 10^−7^ were considered significant. The same parameters were also used to conduct GWAS independently for each of the four ADME populations of our study, and also with a jackknife procedure removing a different population each time, in order to test the sensitivity of the main GWAS to a population effect. Analysis of GWAS results and prioritization of candidate genes were done with the FUMA (Functional Mapping and Annotation) web platform (Watanabe et al. 2017, supplementary text, Supplementary Material online).

### Genomic Scans of Selection

We compared the two most differentiated populations for each of the evaluated phenotypes to identify genes potentially under selective pressures related to the metabolism of exogenous compounds, using the XP-EHH method (Sabeti et al. 2007) and the PBS method (Yi et al. 2010, supplementary text, Supplementary Material online). We also searched for the possible signals of soft sweeps or balancing selection in the four populations using the iHS method (Voight et al. 2006, supplementary text, Supplementary Material online).

## Supplementary Material

evac167_Supplementary_Data

## Data Availability

The pseudonymized genotype and phenotype data, and the covariates used in the GWAS, are deposited in the institutional permanent archive of the University of Geneva (https://doi.org/10/gq568g), where all necessary information for data access can be found (the data will be made available upon signing a Data Use Agreement, assuring that the data will only be used in accordance with the restrictions of the informed consent, and agreeing that the data will be destroyed after the research is complete). All results needed to evaluate the conclusions in the paper are available in the main text or the Supplementary material online, including supplementary figs. S21 to S24.
