# Supplementary material for: Joint Analysis of Phenotypic and Genomic Diversity Sheds Light on the Evolution of Xenobiotic Metabolism in Humans
Source: Genome Biol Evol. 2022 Nov 29;14(12):evac167. doi: 10.1093/gbe/evac167 (PMC9750130; doi:10.1093/gbe/evac167)
Supplement: evac167_Supplementary_Data [file evac167_supplementary_data.zip › Mouterde_SupplementaryTextANDFiguresS1-S24_GBE2022_v4.pdf]

Supplementary Information

Joint analysis of phenotypic and genomic diversity sheds light on the evolution of xenobiotic metabolism in humans

Médéric Mouterde, Youssef Daali, Victoria Rollason, Martina Čížková, Anwar Mulugeta, Khalid K. Al-Balushi, Giannoulis Fakis, Theodoros Konstantinidis, Khalid Al-Thihli, Marie Cerná, Eyasu Makonnen, Sotiria Boukouvala, Said Al-Yahyaee, Getnet Yimer, Viktor Černý, Jules Desmeules, and Estella S. Poloni

This PDF file includes:

- Supplementary text (Supplementary Text on Materials and Methods and Supplementary Text on Results)
- Supplementary Figures 1 to 24
- Supplementary references

Other supplementary materials for this manuscript:

- Supplementary Tables S1 to S25, are provided as separate files in spreadsheet format (.xlsx)

Supplementary Information Text

Supplementary Text on Materials and Methods

Ethical clearance

Before initiation of sampling, the study protocol (deposit ID NCT02789527, at clinicaltrials.gov) was approved by the Ethics Commission of the Canton of Geneva, Geneva (Switzerland), the Institutional Review Board of Charles University, Faculty of Sciences, Prague (Czech Republic), the National Research Ethics Review Committee and the Food, Medicine and Healthcare Administration and Control Authority of Ethiopia, Addis Ababa (Ethiopia), the Medical Research and Ethics Committee of Sultan Qaboos University, Muscat (Sultanate of Oman), and the Research Ethics Committee of Democritus University of Thrace (Greece).

Samples

Healthy volunteers were recruited at academic institutions in the four countries, namely at Addis Ababa University in Addis Ababa (ADD), Sultan Qaboos University in Muscat (MUS), Democritus University of Thrace in Alexandroupolis (ALE), and Charles University in Prague (PRA), using the inclusion/exclusion criteria described below (Figure 1). Each participant provided a saliva sample to be used for DNA extraction and three peripheral blood samples of 10 µL to quantify drug metabolism after ingestion of the Geneva cocktail containing seven probe compounds (1-3). In total, 367 individuals were sampled. Adverse events were recorded as per the approved Study Protocol of the Competent Ethics Committee of each country. An adverse event is defined as an untoward medical occurrence in a participant administered a pharmaceutical product and which does not necessarily have a causal relationship with the study procedure. An adverse event can therefore be any unfavorable and unintended sign, symptom, or disease temporally associated with the use of a medicinal (investigational) product, whether or not related to the medicinal (investigational) product. Adverse events are described in Rollason *et al.* (2), and were categorized as mild to moderate, non-serious, and resolved spontaneously.

Inclusion/exclusion criteria

In each sampling site (Figure 1A), a call for recruitment of approximately 100 healthy volunteers among students and staff was organized, with the following inclusion criteria: participants gave written consent, were aged between 18 and 50 years, had no medication or pathological condition, had two parents and at least three grand-parents from the population (i.e. they were born in the population, see Figure 1B, and had maternal language listed in the country's official languages), fasted and avoided drinking caffeine-containing beverages overnight. As per the approved Study Protocol of the Competent Ethics Committee of each country, inclusion was decided and communicated by the MD (either the PI or the Delegate MD of the local PI) in charge of participants' interviews before entering the sampling site. In particular, based on the MD's decision, individuals with a recent antihistamine or thyroid medication (but stopped for at least 72 hours before sampling), or with a history of thyroid pathology could be included.

Samples' ethnic composition

Each participant to the study informed on her/his birthplace and mother tongue, as well as those of her/his relatives (the two parents and four grandparents). The birthplaces of the four grandparents of all participants in the study are displayed on a map in Figure 1 (panel B).

In Ethiopia (Addis Ababa University sampling site), the sampled individuals and their relatives had all an Afro-Asiatic language as mother tongue (100%), 80% of which had Amhara (i.e. Semitic branch of Afro-Asiatic), but this proportion dropped below 60% or less in their relatives (Supplementary Figure 22). The most represented Afro-Asiatic branch is Semitic

(88.5% of participants, 75% of parents, 72.7% of grandparents), followed by Cushitic (7.7%, 19.7% and 21%), then by Omotic (3.8%, 5.8% and 6.3%). To note that although the participants had all relatives speaking an Afro-Asiatic language as mother tongue, none had all her/his relatives speaking the same mother tongue. Indeed, only about half of the Ethiopian sample consists of individuals, both of whom parents and four grandparents have the same mother tongue, and almost one eighth of the sample includes individuals whose parents' mother tongues are different from their own.

In Oman (Sultan Qaboos University sampling site), all participants were born in the country, and themselves as all their relatives had Arabic as mother tongue. One participant had all relatives with Omani ancestry but born in Tanzania (in the former Sultanate of Zanzibar, which lasted until 1964 and extended over the East African Swahili coast), and one participant had a grandmother from Bahrain.

In Greece (Democritus University of Thrace in Alexandroupolis, sampling site), 98% of participants had Greek as mother tongue, and this proportion was of 96.6% in their parents, and 96.1% in their grandparents. One participant had Russian as mother tongue, and one had Turkish, but both indicated that Greek ancestry was assumed and transmitted in their respective families. Indeed, both families were from the Pontus region, on the southern coast of the Black Sea, which housed a large Greek community until 1923.

In the Czech Republic (Charles University in Prague sampling site), one participant had Slovak as mother tongue, as six additional participants had Slovakian relatives (from one up to six relatives). This was expected, however, given that Czechia and Slovakia were part of former Czechoslovakia, and separated in two independent countries as recently as in 1993.

Geneva cocktail

The Geneva cocktail (1, 2) is designed to measure the activities of six cytochrome P450 enzymes, namely *CYP1A2* (with caffeine, 50 mg), *CYP2B6* (bupropion, 20 mg), *CYP2C9* (flurbiprofen, 10 mg), *CYP2C19* (omeprazole, 10 mg), *CYP2D6* (dextromethorphan, 10 mg), and *CYP3A4* (midazolam, 1 mg), as well as the transporter activity of the P-glycoprotein encoded by *ABCB1* (P-gp, with fexofenadine, 25 mg). For simplicity purposes, metabolic phenotypes are interchangeably reported either with reference to the tested compound (e.g. caffeine) or to the gene targeted by the measured enzymatic function (e.g. *CYP1A2*).

Acquisition of phenotypic data

Collection times of blood samples were at +2, +3 and +6 hours after ingestion of the cocktail. The samples were collected with the HemaXis DB10 Whole Blood device (DBS system SA, Geneva, Switzerland) following the manufacturer's instructions, and stored at ambient temperature in the field and at -20°C in the lab. Drugs and their corresponding metabolites were quantified with LC-MS/MS at the Laboratory of Clinical Pharmacology of the Geneva University Hospitals, as described in Bosilkovska *et al.* (3).

In total, out of the 367 samples of dried blood spots, 365 were successfully quantified for the Geneva cocktail's substrates and metabolites, and were used in downstream data processing (Supplementary Table 24). Concentrations of both substrates and their metabolites, measured at +2, +3 and +6 hours after administration of the probe drugs, were used to calculate areas under the curve (AUC), including a reference concentration of 0 ng.mL^{-1} at +0 hours. An individual phenotype (i.e. level of enzymatic activity) was defined as the $AUC_{metabolite}$ / $AUC_{substrate}$ ratio, and used in following analyzes. The more precise and reliable measurement of AUC ratios was preferred to the traditional method of using the single +2h point in time metabolic ratio (MR), but the interpretation remains the same, i.e. the higher the ratio, the faster the enzymatic activity detected. Note that for the activity associated with the *ABCB1* gene, which encodes a transporter, no ratio was calculated and only the concentration of the P-gp substrate (fexofenadine) was measured. Therefore, the higher the AUC generated for fexofenadine, the slower the transporter activity was. Note that both the low caffeine concentration in the Geneva cocktail (50 mg) and the low blood volume collected (10 μL at each time-point) prevented measuring arylamine N-acetyltransferase activity with LC-MSMS.

Definition of discrete classes of phenotypes

The method for calculating AUC ratios was applied to the cohort of Bosilkovska *et al*. (1) to define thresholds between poor, extensive and rapid metabolizers (Supplementary Figure 23), except for midazolam (*CYP3A4*) for which the traditional MR method at +2h was used (see below). The cohort study by Bosilkovska *et al*. (1) was used to arbitrarily define the lower and upper thresholds, allowing classification of individuals into one of the three phenotypic categories (poor, extensive, rapid) of metabolizers (Supplementary Table 1, Supplementary Figure 23): those thresholds were set approximately midway of the first and third quartiles of the normal and inhibited responses, respectively, for the lower threshold (i.e. between poor and extensive metabolizers), and of the third and first quartiles of the normal and induced responses, respectively, for the upper threshold (i.e. between extensive and rapid metabolizers). Note that no distribution under induction was available for *CYP2D6* and *CYP3A4*, because no inducer is known for *CYP2D6*, whereas for *CYP3A4* the midazolam concentrations under induction were below the detection thresholds of LC-MS/MS. The healthy volunteers phenotyped in this study were each assigned to one of these three discrete categories on the basis of their corresponding AUC ratio or MR for *CYP3A4*.

Sensitivity analyses of phenotypes

For each phenotype, we performed a series of sensitivity analyses by using Kruskal-Wallis tests to compare the populations on subsets of the data, i.e. considering females and males separately, considering only females not on contraceptive pill, considering only non-outlier individuals in the range of ages and of BMIs, considering only non-smokers or non-drinkers (and khat non-chewers - a single individual reported to chew khat, and also drink alcohol; these two factors were thus considered together), and considering only individuals with no medical history, namely no history of antihistamine treatment or thyroid pathology/medication.

Genome-wide genotypes

Saliva samples were collected and stored in DNA Genotek Oragene tubes at ambient temperature in the field and at -20°C after that in the lab. DNA was extracted and purified with the DNA Genotek prepIT.L2P extraction protocol, following the manufacturer's instructions, and quantification was performed with a Qubit 3.0 Fluorometer to ensure the presence of enough material to perform genotyping. Purified DNA was processed at the University of Geneva sequencing facility (iGE3 genomics platform) with an Illumina CoreExome24 array including 555,356 built-in markers that we further enriched with 4,000 custom markers (Supplementary Table 25), of which a subset was chosen for a study of central pain sensitization (4). In total, out of the 367 samples of DNA collected, 358 were successfully genotyped.

The data, generated in Illumina proprietor format, was first processed with the Illumina home software GenomeStudio before being converted to the more easily handled and commonly used PLINK format (5). This step and subsequent quality controls were performed following the protocol of (6) which consists of different filters by individuals (relatedness, gender mismatch, genotype consistency, heterozygosity), markers (SNPs clustering, HW equilibrium) or population (consistency when compared to other datasets). The resulting final dataset, referred to as the ADME dataset, was composed of 352 genotyped DNA samples for 550,416 markers: the results from 4,940 markers were removed because of call rate below 98% and six DNA samples were removed because of call rate below 95% or suspicion of cross contamination. As a control dataset, we also obtained, with the same array and through the same quality controls, the genotypes of 550,416 markers in 119 DNA samples from the Senegalese Mandenka population.

To check if any problems could have arisen in our ADME dataset during the processing of DNA samples or quality controls, and to investigate the structure of this newly generated dataset in comparison to the known genomic structure of the human population, we contrasted it with the 1000 Genomes (1KG) project dataset (7). The whole 1KG dataset was downloaded in VCF format from the 1KG FTP server and converted into PLINK format with VCFTools (8). The two datasets (ADME and 1 KG) were then merged using PLINK, based on the Illumina name of each variant. Variants annotated as tri-allelic (or more) were flipped and

the remaining unmergeable variants were discarded. Similarly, palindromic SNPs were also discarded to avoid strand problems when merging these datasets. Finally, from the 1KG dataset, only population samples from Africa, Europe and Asia were retained. The resulting merged genomic dataset used for population structure analyses thus included the genotypes at 253,526 markers of 2,374 individuals from 24 populations: 19 from the 1KG project and five from this study, i.e. the four genotyped samples of this study (ADME dataset) and the Senegalese Mandenka (SEN). The markers were then filtered on linkage disequilibrium (LD) with PLINK, using overlapping windows of 50 markers per window, 10 markers to shift to the next window, and $r^2 < 0.57$.

As a quality check, a principal component analysis (PCA) was performed with the R package adegenet (9) on the genomic dataset generated in this study, namely the four ADME samples, and the SEN (Supplementary Figure 20). The ADME populations are differentiated mainly by latitude, with the first component representing almost 10% of the total variance. As expected for a Sub-Saharan African population sampled using nationality as the main criterion, the Ethiopians display the largest dispersion in the PC plot, in comparison to the European and Mandenka populations. The Omanis also display a large variation, somewhat at odds with a high level of consanguinity expected in this population due to the widespread practice of endogamous marriages. It is however consistent with the recent history of the Omani Empire, which extended its influence over the Indian Ocean (i.e. the East African, Yemeni and Iranian coasts) around the XVIIIth century, thus possibly facilitating substantial gene flow between populations under Omani governance.

We also performed a second PCA with the same five populations (i.e. the four ADME and SEN) and the 19 1KG populations (Figure 1B). As expected with the use of the 1KG reference panel, the first PCA component highlights a genomic differentiation between African populations and European plus Asian populations, while the second component discriminates populations in Eurasia along longitude. The four populations of our ADME dataset fill the gap between African and European populations, and their position in the PCA plot is consistent with geography: Czechs and Greeks are located among the other Europeans of 1KG, and Ethiopians and Omanis are located between the sub-Saharan African (which also includes the Mandenka) and European clusters. We observe an extended dispersion of Omani samples between the Eurasian and African clusters, highlighting substantial genomic heterogeneity in this population. To note that to account for population structure, the five first principal components of this PCA were included as covariates (along with age, sex, and BMI) in all GWAS analyses.

Finally, we used the ADMIXTURE software to perform an analysis of structuration of our dataset (10). Values of K from 1 to 10 were tested, with 50 iterations per model, and the results of the cross-validation criterion analysis (Supplementary Figure 24) were used to choose the model representing the dataset at best (K = 4, Supplementary Figure 21). Each of the four clusters highlighted by this analysis corresponds to one of the four different “regions” that we sampled, namely West Africa, East Africa, the Middle-East and Europe. Some clusters are found in several populations, and most probably represent gene flow among them (or among un-sampled neighboring populations) rather than admixture events.

Imputation of genotypes for GWAS

New markers were imputed from our genotyping data with the Michigan Imputation Server (MIS), using Minimac4 (11), following the instructions provided by MIS for data preparation and pre-imputation quality control. The PRA and ALE datasets were combined into a single European dataset (EUR) for imputation. Rsq was set to 0.3 and 1KG was chosen as a panel reference with 1KG AFR for ADD, 1KG EUR for EUR and 1KG Mixed for MUS as reference populations. After quality control using the data from 1KG as a comparison, 297,692 markers for ADD, 341,975 for EUR and 327,982 for MUS, respectively, were retained. The data was phased with Eagle v2.4 (12). After imputation, these three datasets were merged and markers were filtered for minor allele frequency (MAF) > 0.05, leading to a total of 5’773’125 markers.

Functional Mapping and Annotation

We used the FUMA (Functional Mapping and Annotation) web platform (13) to map and analyze the results of the GWAS. This platform allows the take up of summaries of GWAS results to annotate candidate markers and prioritize candidate genes using different methods (MAGMA, gene position mapping, eQTL mapping and 3D chromatin mapping).

MAGMA analysis consist of three different approaches: a gene-based GWAS in which SNPs are assigned by their locations to a gene and a single combined *p-value* is computed, a gene-set analysis in which genes are grouped according to GO pathways so as to identify pathways to prioritize, and a tissue expression analysis in which genes are grouped according to specific tissue expression categories.

FUMA parameters

FUMA detects and prioritizes different regions according to the genomic position of candidate markers (gene position mapping), the influence on the expression of certain traits (eQTL mapping), and the influence on the 3D interaction of chromatin (3D chromatin mapping). Candidate markers were defined using LD, namely $r^2 > 0.6$ with the lead marker, and markers with $r^2 > 0.1$ were defined as part of the same locus. The maximum distance between of the LD blocks to merge was set to 250 kb.

For gene mapping, the distance between markers and a gene was set to 10 kb. For eQTL mapping, the eQTLgen, Blood eQTL, Blood BIOS QTL and the GTEx databases were used; blood, adrenal gland, brain, liver, lung, skin, small intestine, and testis tissues were selected for the GTEx databases, and the threshold was set at a false discovery rate (FDR) < 0.05. For the 3D interaction of chromatin, the databases used from the HI-C database were Adrenal, Liver, and Lung, the FDR threshold was set to 1e-6, and the windows for promoter regions were set at 250 bp upstream and 500 bp downstream.

For each mapping, candidate SNPs were only kept if their associated CADD score was ≥ 12.37, which is the threshold for which a marker is considered as deleterious, or if RegulomeDB score was < 2, which indicates a high probability of at least affecting binding. Prioritization of genes was restricted to protein coding genes.

Genomic scans of selection

In order to identify genes potentially under selective pressures related to the metabolism of exogenous compounds, we performed genomic scans according to three alternative approaches.

First, we used the XP-EHH method (14), which exploits the extent of homozygosity on haplotypes to detect genomic areas under selection by comparing two populations. In our case, the aim was to compare, for each of the phenotypes, the two populations with the most extreme phenotypes, in order to identify candidate genomic zones liked to the difference in metabolic rates. Since the power of the analysis is improved with phased data, we again used the Michigan Imputation Server to phase our data with Eagle ver2.4 using recommended parameters (12). The phased data included 312’323 markers and was analyzed with the R package *rehh* (15). The identification of candidate areas was performed using 200 kb non-overlapping windows, and any window containing at least one marker presenting an XP-EHH statistic within the 0.01% of extreme values of the distribution of XP-EHH scores was declared as candidate zone.

Second, we used the population branch statistic (PBS), which identifies, in a target population, those sites that underwent a stronger variation in allelic frequency, hence in F_{ST}, than expected from the empirical distribution of genome-wide pairwise F_{ST} values between the target population and each of two reference populations (16). The target and first reference populations were chosen according to the evaluated phenotype (as in the XP-EHH tests), whereas the Senegalese Mandenka (SEN) were chosen as the second reference population. We used PLINK (5) to obtain genome-wide per-site pairwise F_{ST} values on all autosomal markers (515,676 markers), and applied a sliding-window approach with overlapping windows of 20 markers and a step size of 5 markers, as in (17). The resulting distribution of PBS window values was visualized with the qqman package of R (18). We set

the detection threshold at the 99.99th percentile of the empirical distribution, with at least two consecutive sliding windows with an associated PBS value above this threshold, to declare a genomic region as associated with an extreme F_{ST} change in the target population, and hence potentially indicating that it experienced population-specific positive selection.

Finally, we used the iHS method (19), which similarly to XP-EHH also relies on the extent of homozygosity on haplotypes to detect genomic regions that have potentially experienced directional selection. However, in contrast to XP-EHH, this approach is able to detect softer sweeps, with candidate markers displaying intermediate frequencies. The analysis was performed with the R package *rehh* (14), on the same phased dataset of 312’323 markers as used for the XP-EHH scans. To identify candidate regions, the iHS score was computed in non-overlapping windows of 200 kb and the -log10(*p*-value) was averaged in windows of 10 SNPs. The top 12 regions were declared candidate zones.

Supplementary Text on Results

Analysis of potential confounders in phenotype distributions and GWAS

Supplementary Figures 13 to 19 display the position of recorded variables (or extreme values taken by those variables) in the phenotype distributions categorized either by BMI or by population and sex, namely smoke, alcohol and khat chewing habits, medical condition with respect to antihistamine treatment, thyroid medication or pathology, and adverse events.

Khat consumption was only declared by a single Ethiopian male participant. For two phenotypes, caffeine (CYP1A2) and omeprazole (CYP2C19) metabolism, his AUC ratio was located in the upper-end outlier values of his population and sex category (ADD males), thus inflating the higher values of these distributions and thereby mitigating population differences.

Antihistamine treatment was only documented among seven Czech participants (6 females, 1 male). Two females had AUC values among the five upper-end outlier values of dextromethorphan (CYP2D6), and two others among three upper-end outlier values fexofenadine phenotypes (P-gp). However, for all other participants with a history of antihistamine treatment AUC values were distributed below and above the median of their respective population and sex category, the only exception being for dextromethorphan, for which all seven individuals with antihistamine treatment had an AUC ratio located above the median (thus with a slower metabolic activity than the median). It is thus highly unlikely that antihistamine treatment may have significantly influenced the phenotyping results, consistent with the sensitivity analyses results (see below).

The same conclusion holds for thyroid medication or thyroid pathology, only reported among four female participants (one in Greece, three in the Czech Republic), since their associated AUC values were never located among the outlier values of their respective population and sex category in any of the seven measured phenotypes.

Inspection of Supplementary Figures 13 to 19 indicated that AUC values of individuals with different smoking and alcohol consumption levels, and of female individuals on contraceptive pill, were distributed over the full range of the phenotypes' diversity, and no particular trend was observed for adverse events.

To further investigate the potential influence on phenotype of those variables, as well as sex, age and BMI, we performed a series Kruskal-Wallis tests on subsets of the data, i.e. considering females and males separately, considering only females not on contraceptive pill, considering only non-outlier individuals in the range of ages and of BMIs, considering only non-smokers or non-drinkers (and khat non-chewers - a single individual reported to chew khat, and also drink alcohol; these two factors were thus considered together), and considering only individuals with no medical history, namely no history of antihistamine treatment or thyroid pathology/medication (Supplementary Table S1).

For four phenotypes – metabolisms of caffeine (CYP1A2), bupropion (CYP2B6), flurbiprofen (CYP2C9), fexofenadine (P-gp) – all Kruskal-Wallis tests reproduced the significant results of the full dataset, thereby implying that the differentiated phenotypic distributions between populations shown in Figure 2 (panels A, B, C, and G) of the main manuscript, are significant independently from sex (and contraceptive pill), age, BMI, smoke, alcohol, khat, and medical history.

For midazolam metabolism (CYP3A4), the results of Figure 2 (panel F) indicated a significant difference between Ethiopians and Omanis, that was reproduced when considering only women, but not anymore when considering only women not on contraceptive pill. However, neither in Oman nor in Ethiopia did any female participant take the contraceptive pill. Actually, when running the Wilcoxon test comparing midazolam metabolism in Ethiopian and Omani women, the result was significant before correction for multiple testing (Wilcoxon test, *p-value* = 0.0379), thus indicating that the non-consistent result for this phenotype when considering

only women not on contraceptive pill is due to a loss of power in this data subset as compared to the full dataset.

Still for the midazolam metabolism (CYP3A4) phenotype, Kruskal-Wallis tests indicated that the difference between Ethiopians and Omanis was not significant anymore when considering only individuals below 28 years of age. Since the sample mean (and median) age in Ethiopia was lower than that in Oman, we checked the significance of the comparison of midazolam metabolism between Ethiopians and Omanis by comparing non-outlier individuals with respect to their own population sample, and indeed here the difference was significant (Wilcoxon test, *p-value* = 0.0015). We reproduced this result by comparing all Ethiopian individuals with only those Omani individuals whose age falls in the lower 80% of ages' distributions in that sample (i.e. of age < 32 years, associated *p-value* = 0.0428). These results thus underline a differentiated phenotypic distribution between Ethiopians and Omanis that is significant independently from age (as well as from the other variables considered).

For omeprazole metabolism (CYP2C19), we failed to reproduce the significant difference between populations (Figure 2, panel D) when considering only individuals that do not drink alcohol, but we found a significant difference when considering only non-smokers. These results are thus suggestive of an influence of alcohol consumption on this metabolism. To verify the possible influence of alcohol consumption, we successively excluded individuals drinking on a daily basis, on a weekly basis, individuals from both categories, as well as those drinking only occasionally. The Kruskal-Wallis test *p-values* were, respectively, of 8.7E-07, 9.8E-07, 8.7E-07, and 0.3812. These results thus suggest that occasional drinkers, the majority of which were associated to the Czech sample (the Greek sample could not be phenotyped for omeprazole metabolic activity), significantly contribute to population differences in this phenotype. Actually most outlier values (in the range of rapid metabolizers) are associated to individuals reporting an occasional consumption of alcohol.

To assess if alcohol consumption could affect the GWAS result of omeprazole metabolism (Figure 6), we performed a new GWAS considering this variable as covariate, along with the other variables, namely age, sex and body mass index (BMI), as well as the five first principal components of the PCA. The resulting Manhattan plot (displayed in Supplementary Figure 12) faithfully reproduces the result displayed in Figure 6, consistently supporting the association of the phenotype with the three genomic regions discussed in the main text (*CYP2C19*, *TPM4* *COX6CP16* regions, on chromosomes 10, 19 and 16, respectively).

Finally, for dextromethorphan metabolism (CYP2D6), interpretation of the results associated to data subsets is more complex. We found no difference between populations with the full dataset (Figure 2, panel E), but a significant difference when considering only women (*p-value* = 0.0471) that was not reproduced when considering only women not on contraceptive pill (*p-value* = 0.18). A contraceptive pill was used by 43% of the Czech female sample, 11% of the Greek female sample, and none of the Ethiopian and Omani female samples. To further investigate these results, we used pairwise Wilcoxon tests to compare female population samples once using all women, once excluding women on contraceptive pill. Considering also women on contraceptive pill, two pairwise comparisons were significant at the 5% level without correction for multiple testing, i.e. that of Czech and Ethiopian women, and that of Czech and Omani women (*p-values* of, respectively, 0.015 and 0.031), but not anymore after multiple testing correction (*p-values* of, respectively, 0.09 and 0.16). Considering only women not on contraceptive pill, none of the pairwise comparisons was significant, even before correction for multiple testing, but a loss of power due to reduction in sample size cannot be excluded. Altogether, the results of these comparisons thus suggest that the contraceptive pill could have a significant, albeit small, influence on dextromethorphan metabolism. However, we also observed that three Czech female participants on contraceptive pill have among the slowest dextromethorphan metabolisms, and one has the most slow value for all participants, whereas other female participants on contraceptive pill have a median metabolism or even much faster than the median.

To assess if taking a contraceptive pill could affect the GWAS result of dextromethorphan metabolism (Figure 7), we performed a new GWAS only of female participants, considering this variable as covariate, along with the other variables, namely age, body mass index (BMI),

and the five first principal components of the PCA. Although excluding male participants reduced the sample size to 197, the resulting Manhattan plot (displayed in Supplementary Figure 11) mainly reproduces the result displayed in Figure 7, thus again consistently supporting the association of the phenotype with the genomic region of *CYP2D6*, on chromosome 22.

Results of the tests for equality of proportions of the three discrete phenotypic categories between populations

Proportions of individuals per phenotype category in each population are shown in Figure 3 and Supplementary Table 3. Results of the tests of equality of proportions between populations are shown in Supplementary Table 4.

Significant variation in proportions of rapid and extensive metabolizers was found for bupropion metabolism (CYP2B6). While none of the individuals in any of the four population samples is characterized as poor metabolizer, all populations display significantly different proportions of rapid and extensive metabolizer phenotypes, except for the comparison of Ethiopians vs Czechs.

For fexofenadine transport (P-gp), rapid metabolizers are significantly more frequent in Ethiopians (43%) and poor metabolizers are significantly more frequent in Omanis (48%), respectively, while extensive metabolizer phenotypes are significantly more frequent in the two European populations (82% in Greeks, 79% in Czechs) compared to Ethiopians and Omanis.

In contrast to the analysis with AUC ratios, discrete categorization of caffeine metabolism phenotypes (CYP1A2) only distinguishes the Ethiopian population sample, which includes significantly fewer rapid (9.7%) and significantly more extensive (79.6%) metabolizers than any other of the three population samples.

For omeprazole metabolism (CYP2C19), the only significant difference is a lower proportion of poor metabolizers in Czechs (30.3%).

The proportions of extensive metabolizers are similar among populations for flurbiprofen metabolism (CYP2C9), but in the Omanis, rapid and poor metabolizers were found, respectively, to be significantly more (51%) and significantly less (18%) frequent than in the other three populations.

Similarly to the results with AUC ratios, no significant differences in proportions were found for dextromethorphan metabolism (CYP2D6), but in contrast to the former, no significant differences were observed for midazolam metabolism (CYP3A4) either.

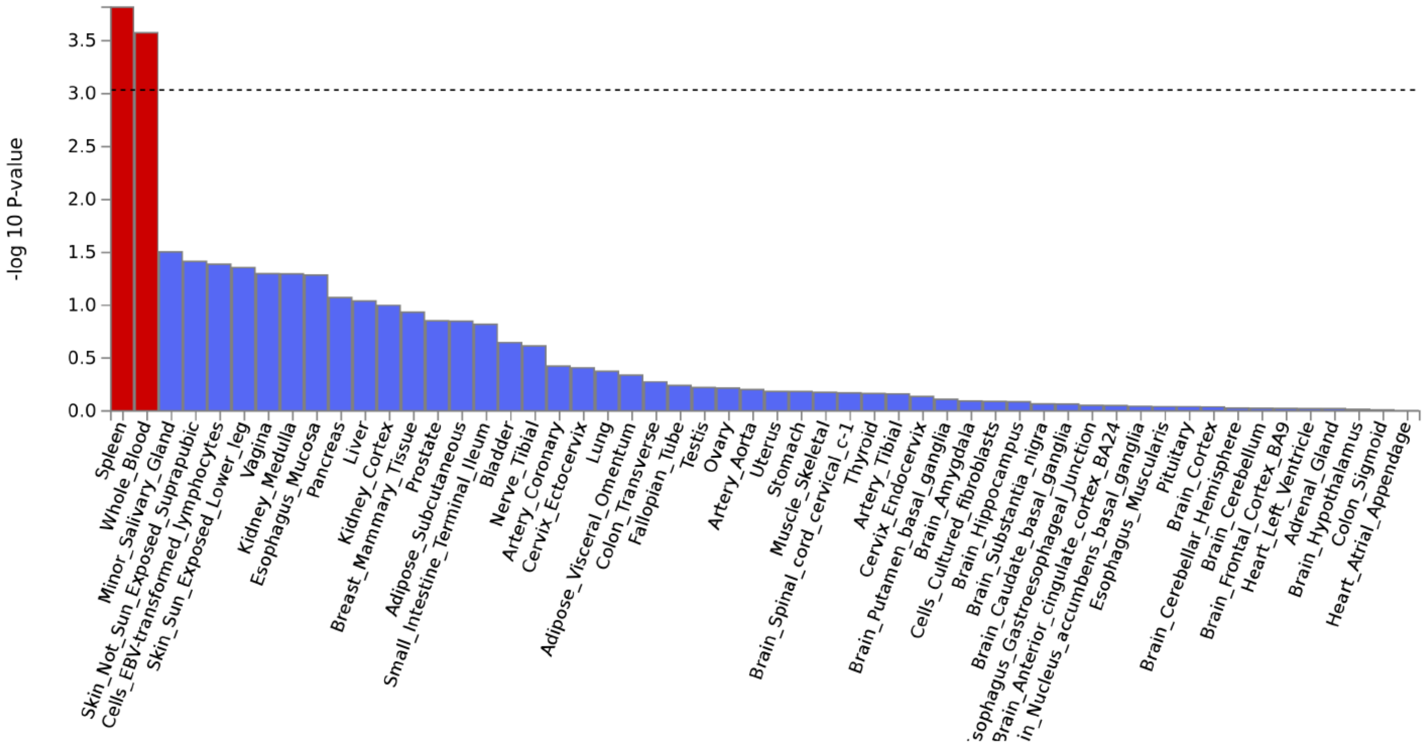

Supplementary Figure 1. MAGMA tissue expression analysis for transformation rate of bupropion. Red bars highlight the two significant associations (blue bars show non significant associations).

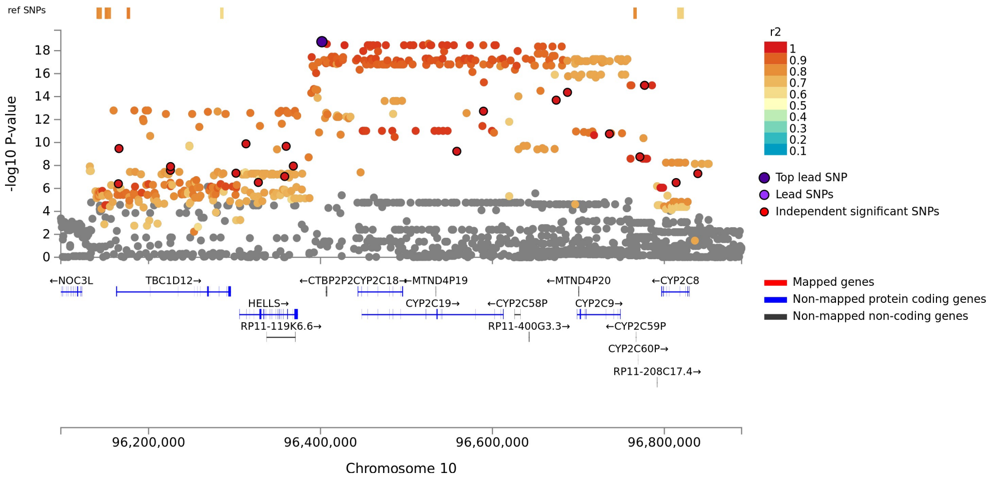

(A)

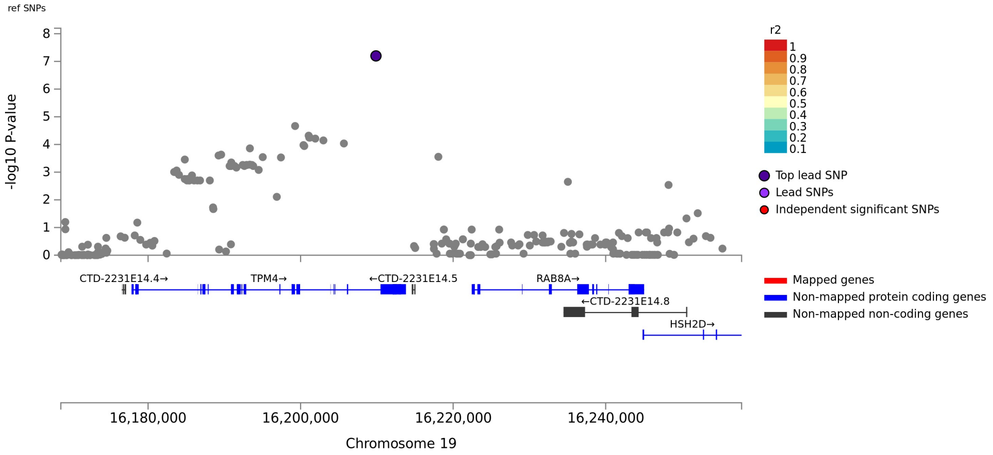

(B)

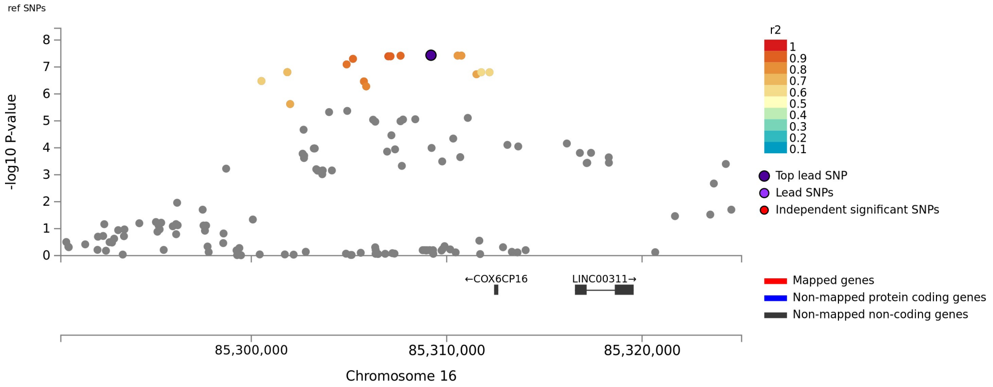

(C)

Supplementary Figure 2. Regional plot of the three FUMA's identified genomic risk loci (GRL) associated with transformation rate of omeprazole. (A) GRL on chromosome 10. (B) GRL on chromosome 16. (C) GRL on chromosome 19.

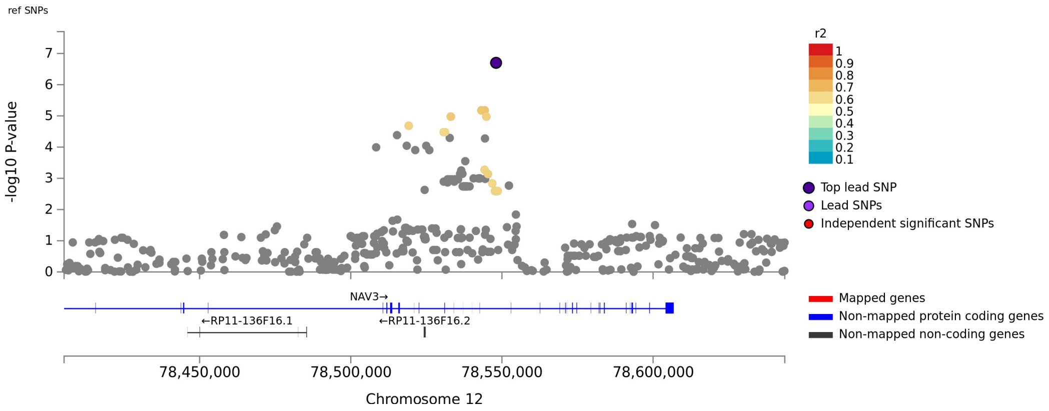

(A)

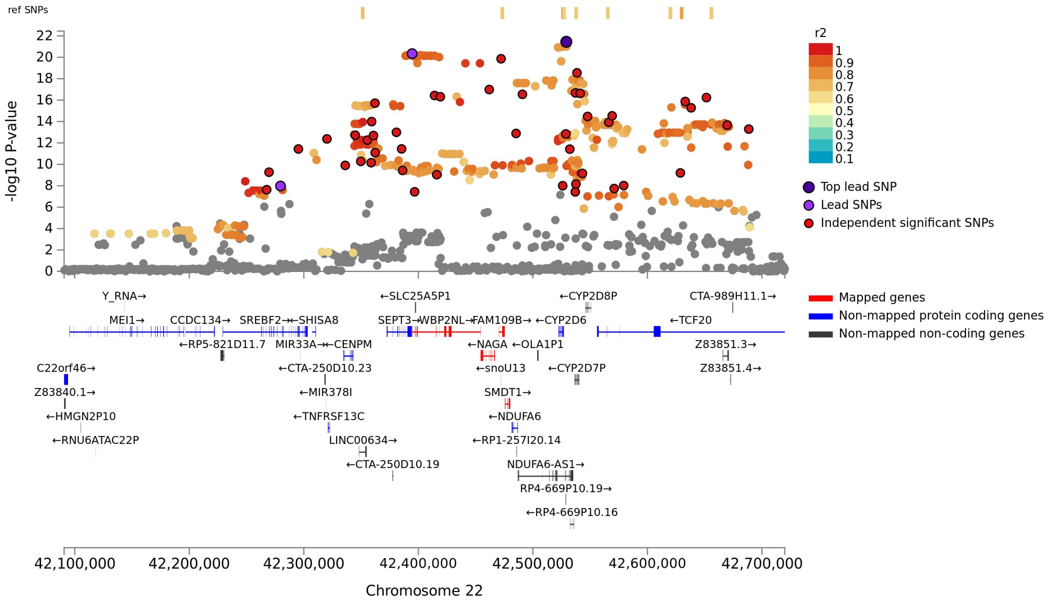

(B)

Supplementary Figure 3. Regional plot of the two FUMA's identified genomic risk loci (GRL) associated with transformation rate of dextromethorphan. (A) GRL on chromosome 12. (B) GRL on chromosome 22.

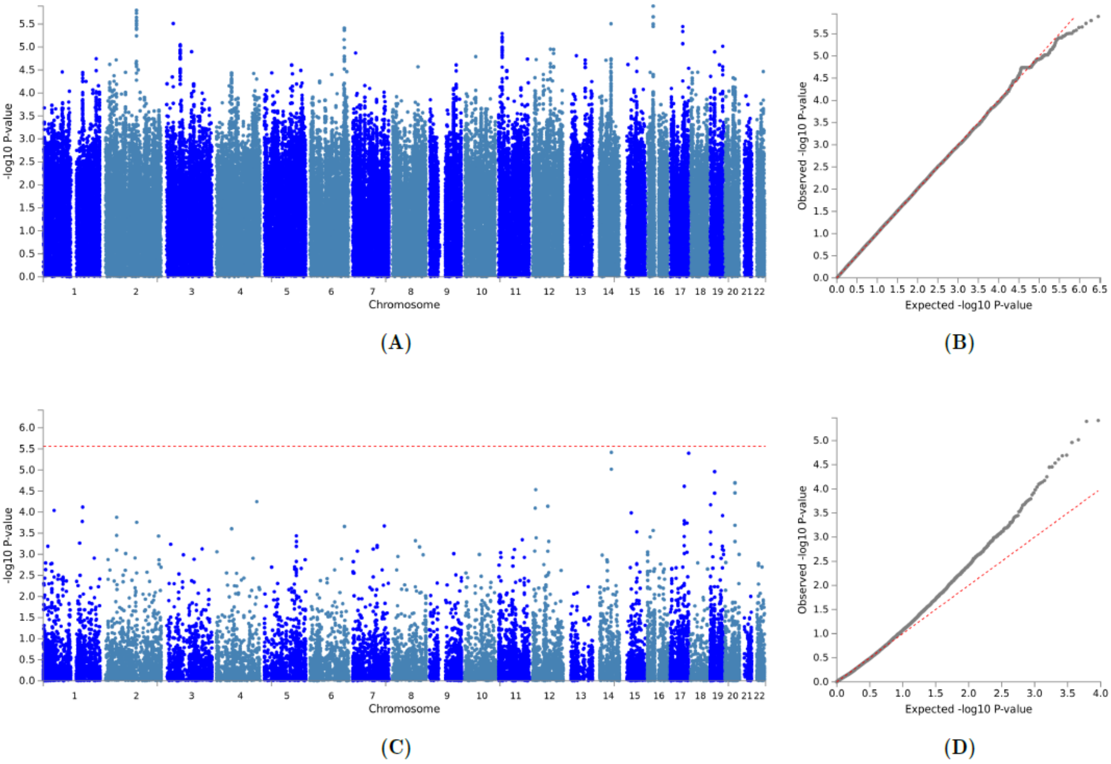

Supplementary Figure 4. GWAS and FUMA gene-based GWAS for transformation rate of caffeine. (A) Manhattan plot of GWAS. (B) Q-Q plot of GWAS. (C) Manhattan plot of FUMA's gene-based GWAS. (D) Q-Q plot of FUMA's gene- based GWAS.

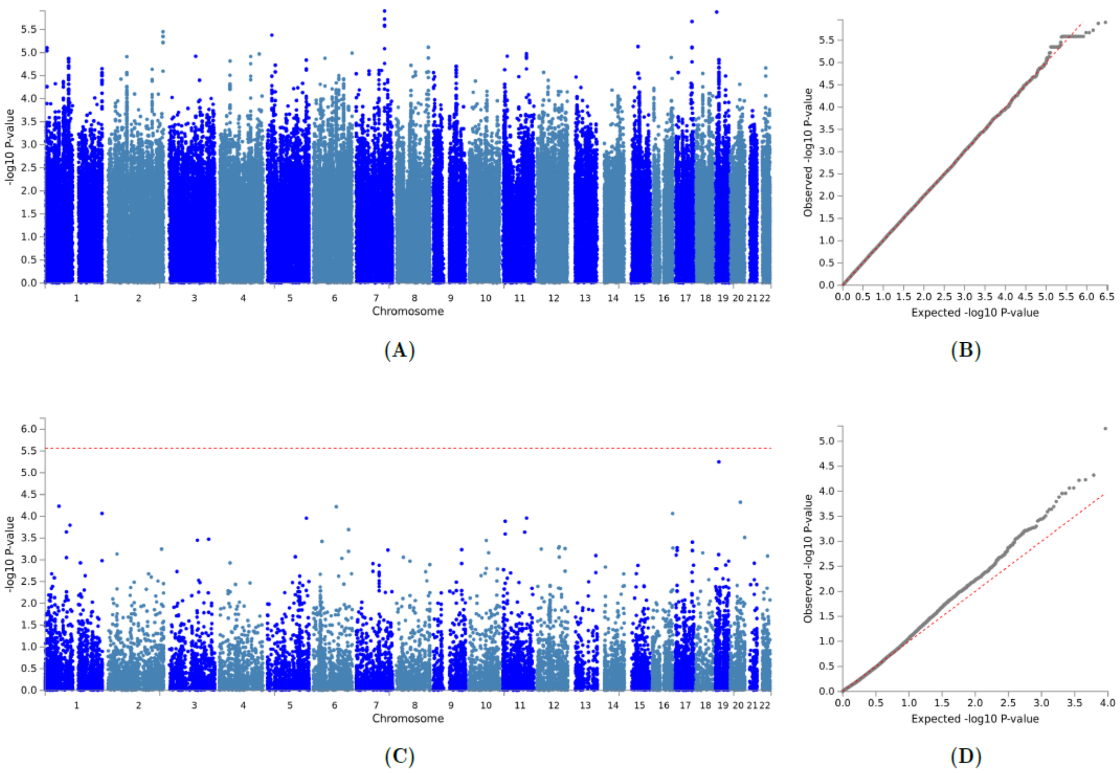

Supplementary Figure 5. GWAS and FUMA gene-based GWAS for transformation rate of midazolam. (A) Manhattan plot of GWAS. (B) Q-Q plot of GWAS. (C) Manhattan plot of FUMA's gene-based GWAS. (D) Q-Q plot of FUMA's gene- based GWAS.

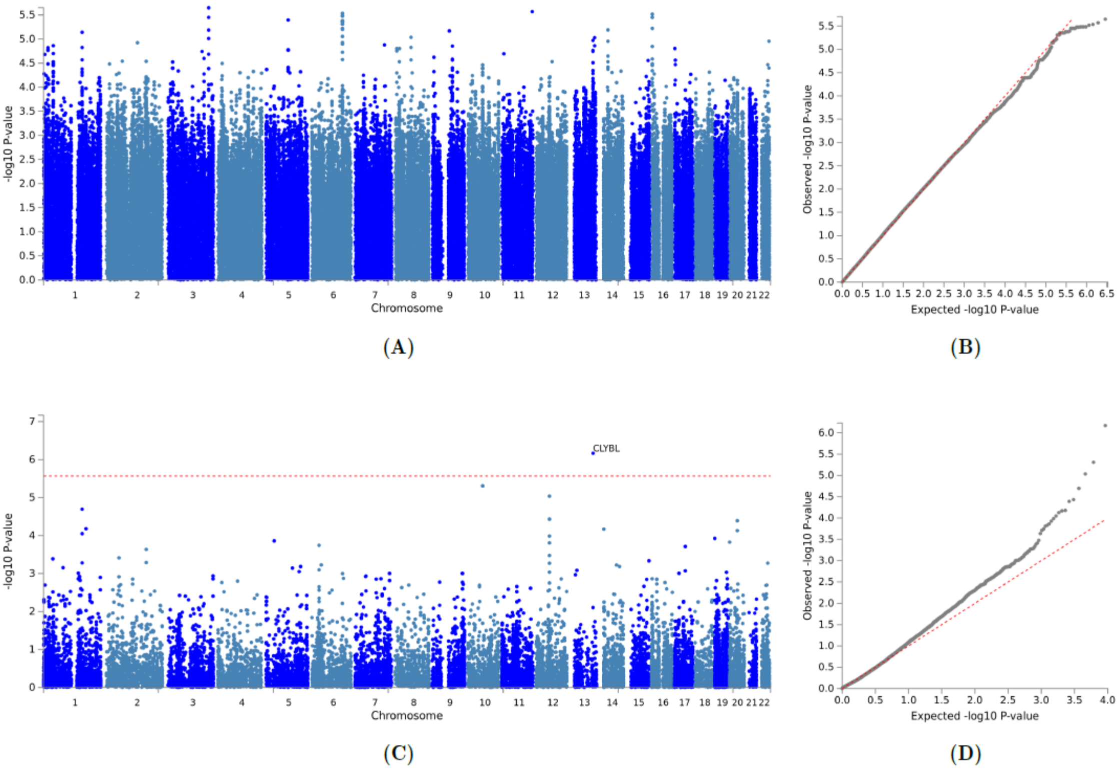

Supplementary Figure 6. GWAS and FUMA gene-based GWAS for transformation rate of fexofenadine. (A) Manhattan plot of GWAS. (B) Q-Q plot of GWAS. (C) Manhattan plot of FUMA's gene-based GWAS. (D) Q-Q plot of FUMA's gene- based GWAS.

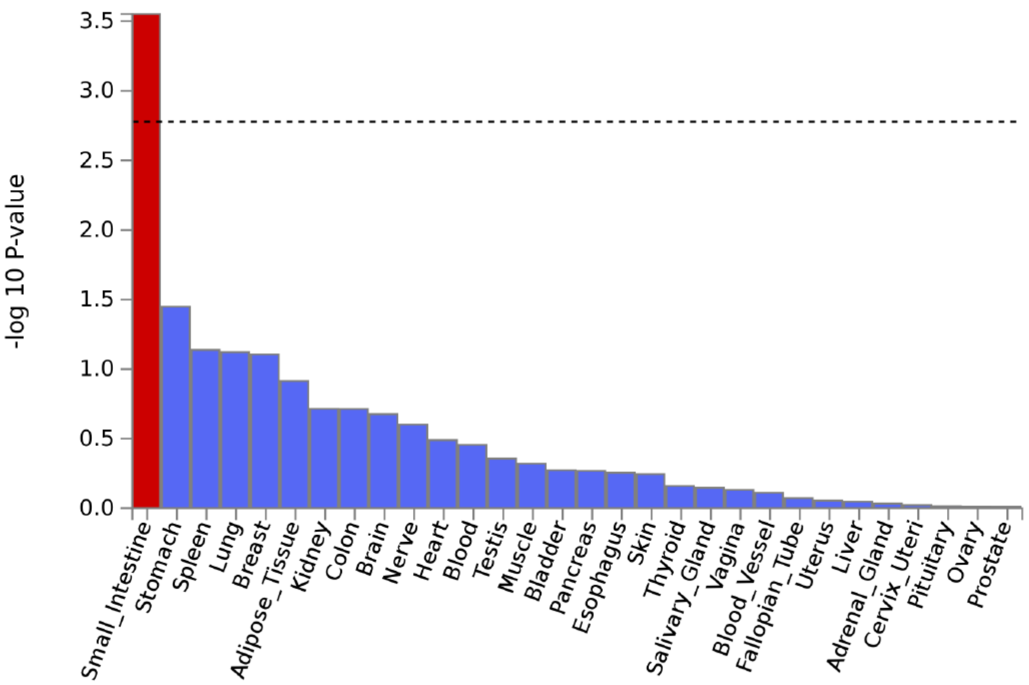

Supplementary Figure 7. MAGMA tissue expression analysis for transformation rate of caffeine. The red bar highlights the single significant association (blue bars show non significant associations).

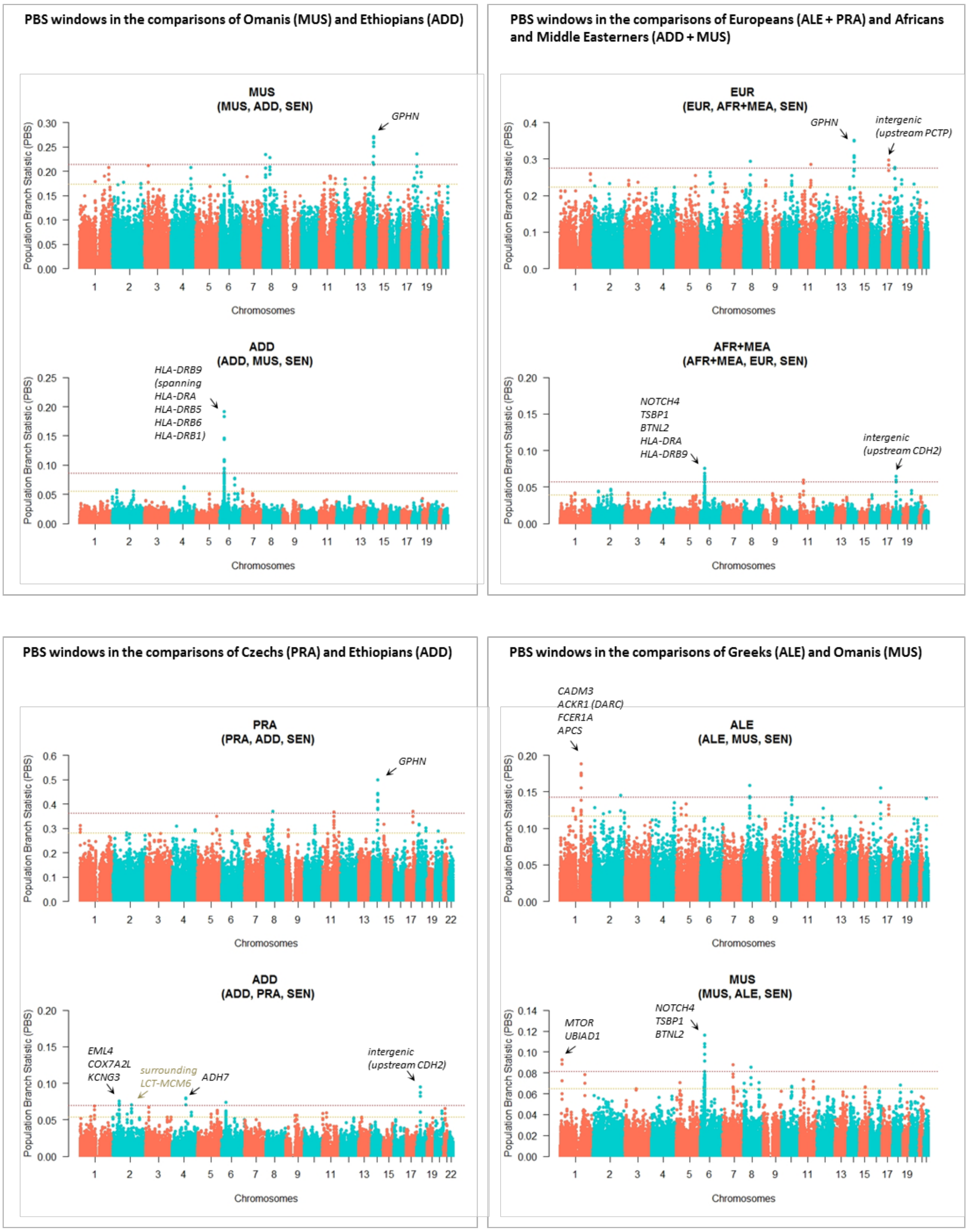

Supplementary Figure 8. Manhattan plots of PBS windows for the comparisons of the two most differentiated populations for each of the evaluated phenotypes, using the Senegalese Mandenka (SEN) as outgroup. The light orange and heavy red dashed horizontal lines represent the values of, respectively, the 99.95th and 99.99th percentiles of the empirical distribution. Genes/genomic regions associated with the highest peaks are indicated by black arrows. For comparison, the light brown arrow indicates the *LCT-MCM6* region.

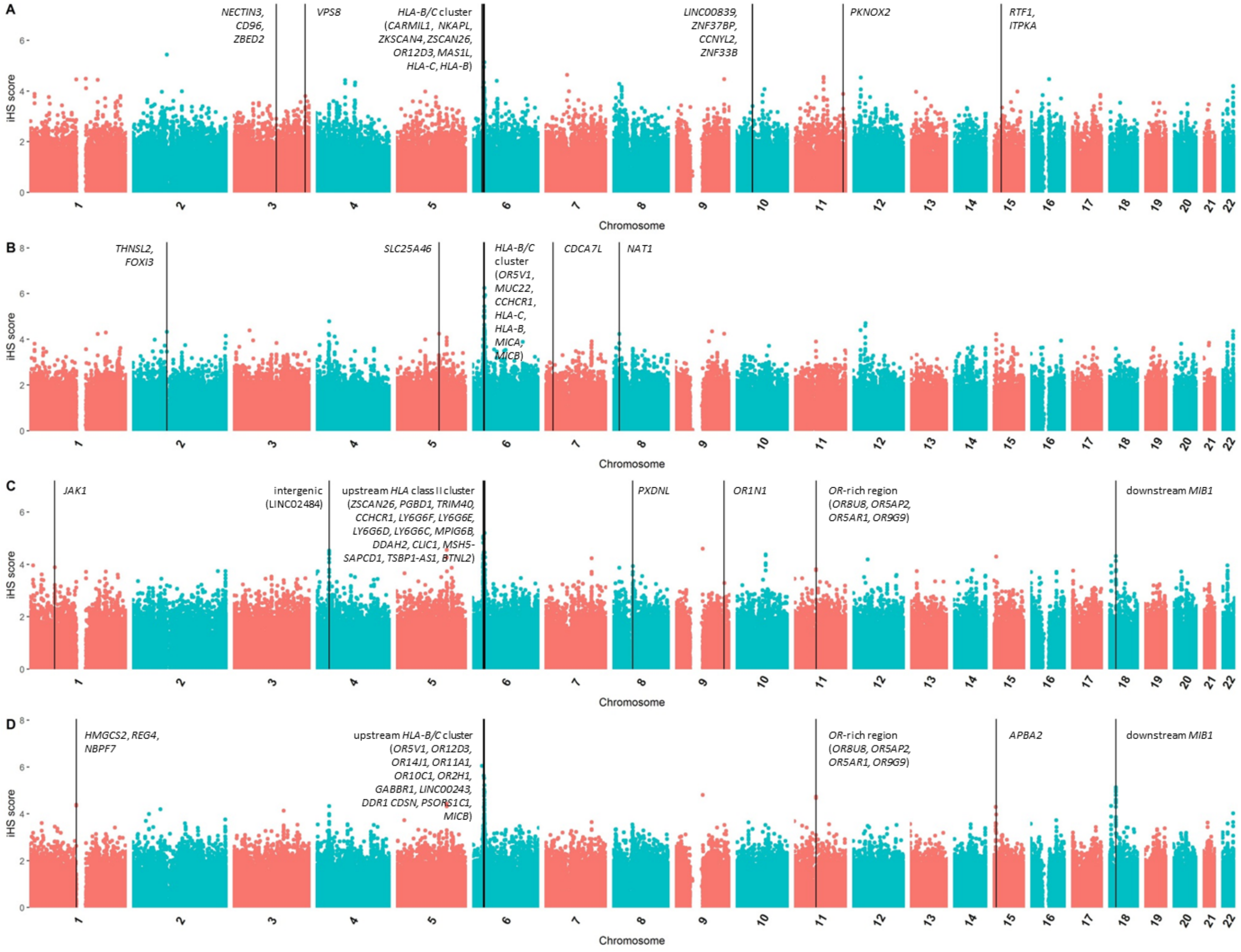

Supplementary Figure 9. Manhattan plot of iHS scores in the four populations tested, respectively, (A) Ethiopians, (B) Omanis, (C) Greeks and (D) Czechs. Genomic regions associated with the highest peaks are indicated by black vertical lines with names of genes indicated besides.

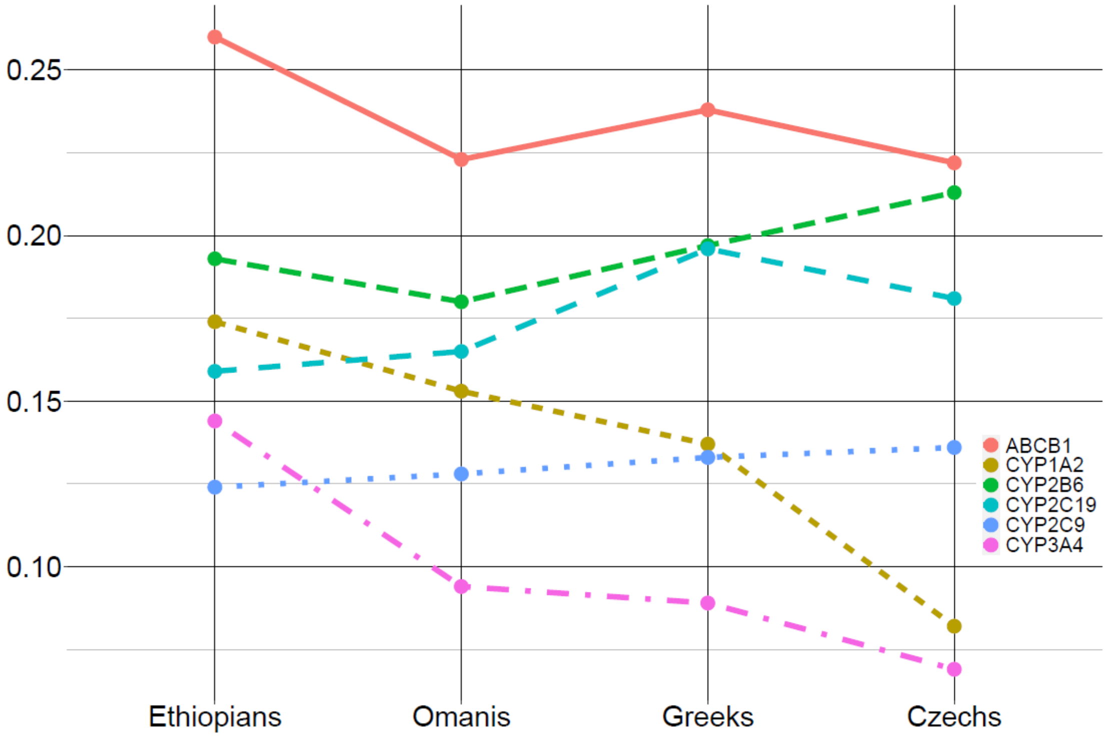

Supplementary Figure 10. Nucleotide diversity estimated from SNPs mapping to six genes targeted by the Geneva cocktail. Nucleotide diversity was not estimated for *CYP2D6*, due to the potentially confounding presence of duplicated gene copies.

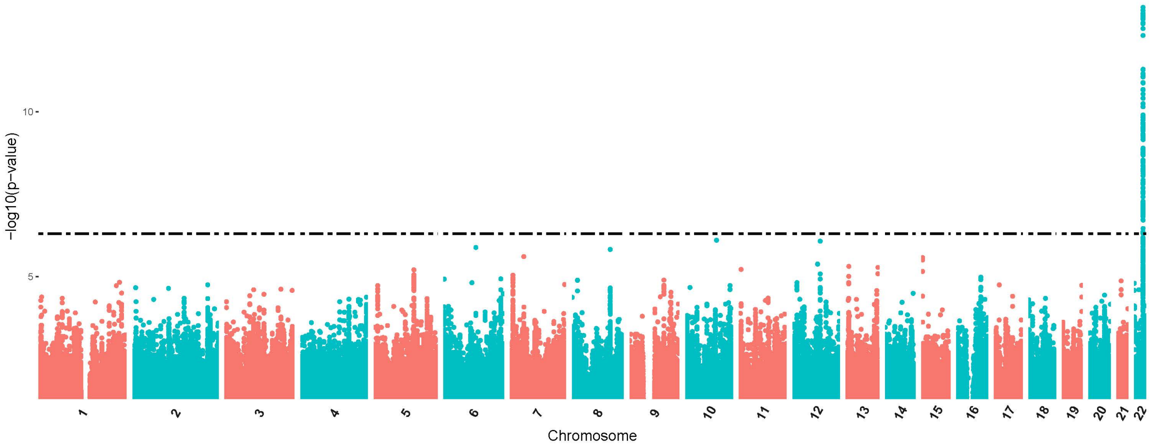

Supplementary Figure 11. Manhattan plot of GWAS for transformation rate of dextromethorphan in women, including, as covariates, age, body mass index (BMI), the five first principal components of the PCA, and use of a contraceptive pill.

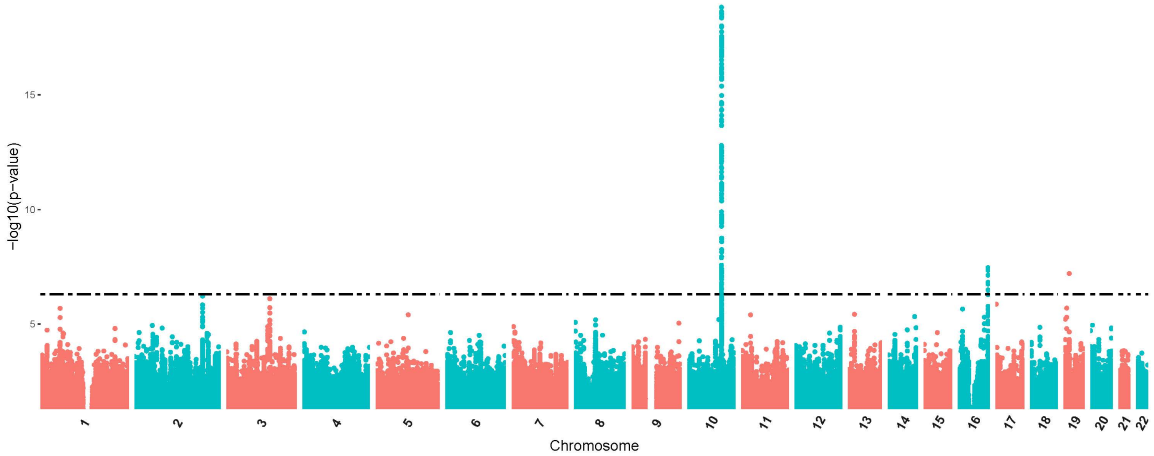

Supplementary Figure 12. Manhattan plot of GWAS for transformation rate of omeprazole, including as covariates, age, sex, body mass index (BMI), the five first principal components of the PCA, and alcohol consumption.

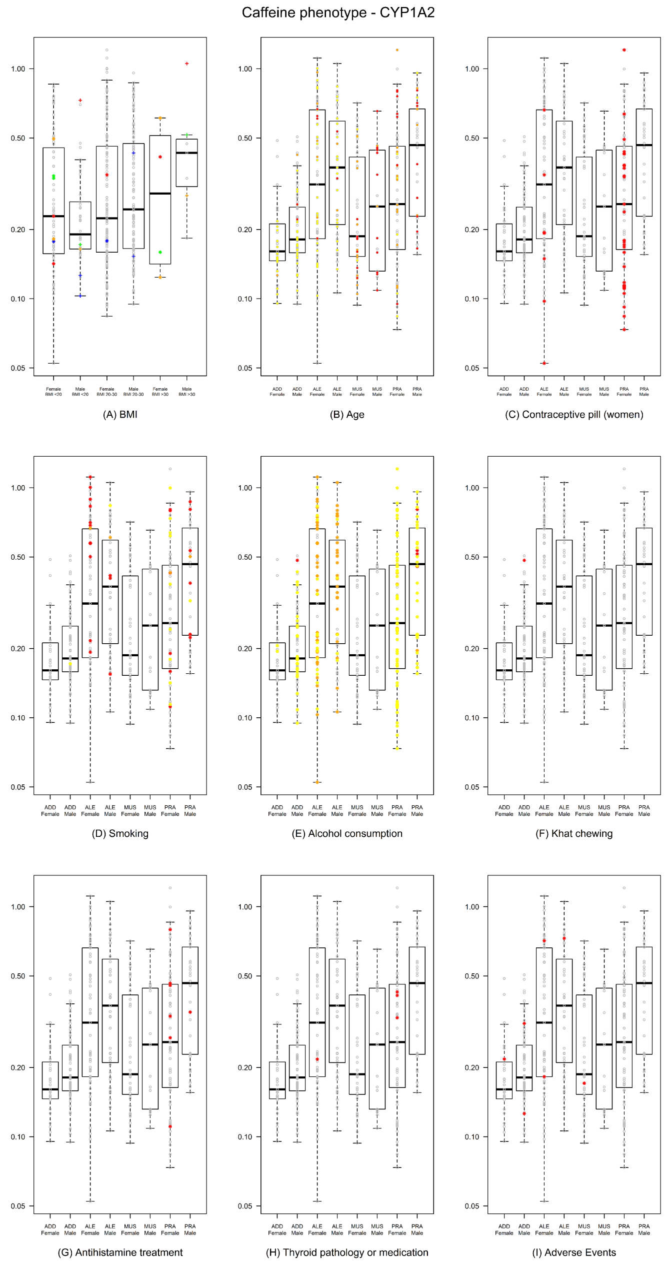

Supplementary Figure 13. Caffeine phenotype distribution (displayed in logarithmic scale) as a function of BMI and sex (A), and of population and sex (B to I). Individual values are shown by gray circle symbols on top of boxplots. In (A) star and plus symbols indicate, respectively, individuals with BMI above or below the 95% of the BMI distribution in their sex category and population (ADD: blue, MUS: orange, ALE: red, PRA: green). In (B) star symbols indicate, respectively, individuals younger than 21 years (< the first quartile of age distribution, yellow), older than 24 years (> the third quartile of age distribution, orange), or older than 28 years (> the 85% of age distribution, red). In (C) red stars indicate women taking contraceptive pill. In (D) and (E) star symbols indicate individuals that, respectively, smoke or drink alcohol daily (red), weekly (orange) or occasionally (yellow). In (F) the red star symbol indicates the individual that chews khat. In (G), (H) and (I) red star symbols indicate, respectively, individuals with a history of antihistamine treatment, of thyroid pathology or thyroid treatment, or that experienced an adverse event during the sampling trial.

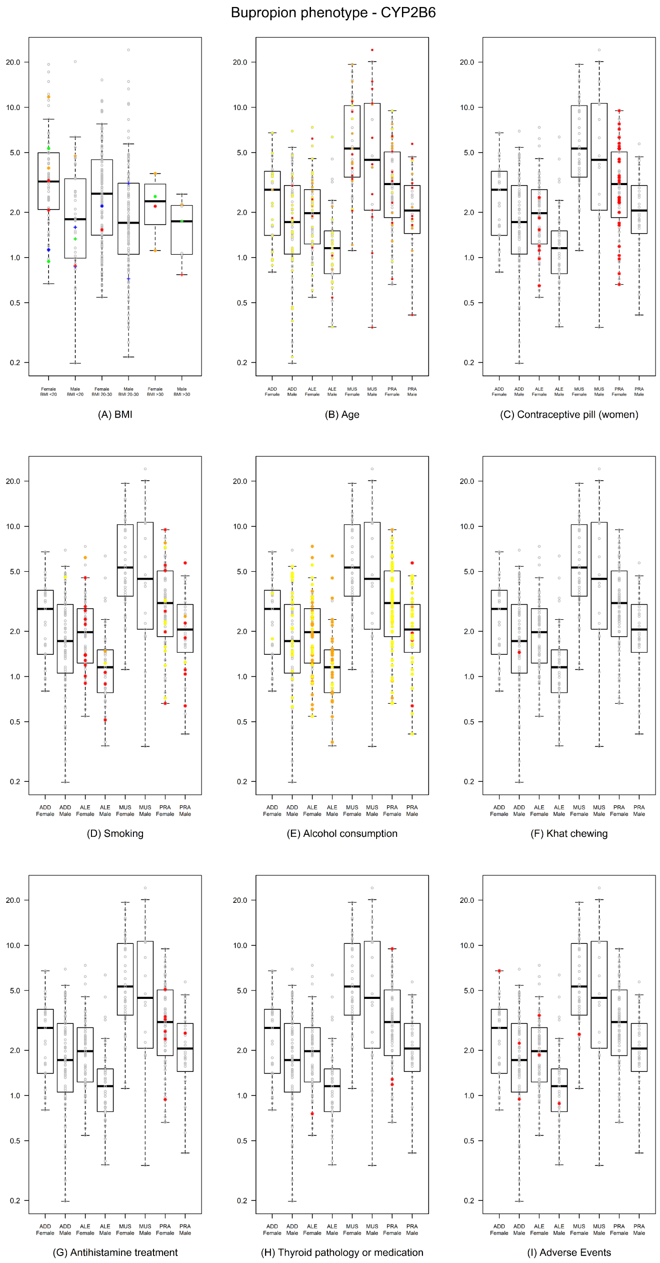

Supplementary Figure 14. Bupropion phenotype distribution (displayed in logarithmic scale) as a function of BMI and sex (A), and of population and sex (B to I). Individual values are shown by gray circle symbols on top of boxplots. In (A) star and plus symbols indicate, respectively, individuals with BMI above or below the 95% of the BMI distribution in their sex category and population (ADD: blue, MUS: orange, ALE: red, PRA: green). In (B) star symbols indicate, respectively, individuals younger than 21 years (< the first quartile of age distribution, yellow), older than 24 years (> the third quartile of age distribution, orange), or older than 28 years (> the 85% of age distribution, red). In (C) red stars indicate women taking contraceptive pill. In (D) and (E) star symbols indicate individuals that, respectively, smoke or drink alcohol daily (red), weekly (orange) or occasionally (yellow). In (F) the red star symbol indicates the individual that chews khat. In (G), (H) and (I) red star symbols indicate, respectively, individuals with a history of antihistamine treatment, of thyroid pathology or thyroid treatment, or that experienced an adverse event during the sampling trial.

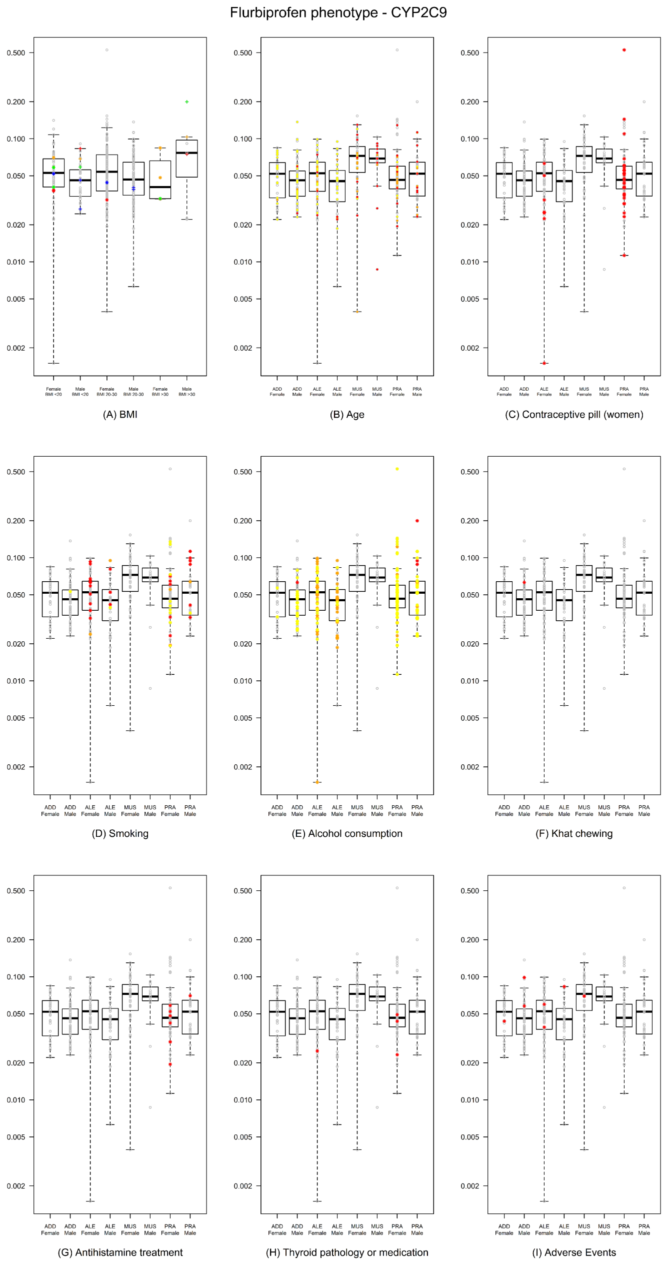

Supplementary Figure 15. Flurbiprofen phenotype distribution (displayed in logarithmic scale) as a function of BMI and sex (A), and of population and sex (B to I). Individual values are shown by gray circle symbols on top of boxplots. In (A) star and plus symbols indicate, respectively, individuals with BMI above or below the 95% of the BMI distribution in their sex category and population (ADD: blue, MUS: orange, ALE: red, PRA: green). In (B) star symbols indicate, respectively, individuals younger than 21 years (< the first quartile of age distribution, yellow), older than 24 years (> the third quartile of age distribution, orange), or older than 28 years (> the 85% of age distribution, red). In (C) red stars indicate women taking contraceptive pill. In (D) and (E) star symbols indicate individuals that, respectively, smoke or drink alcohol daily (red), weekly (orange) or occasionally (yellow). In (F) the red star symbol indicates the individual that chews khat. In (G), (H) and (I) red star symbols indicate, respectively, individuals with a history of antihistamine treatment, of thyroid pathology or thyroid treatment, or that experienced an adverse event during the sampling trial.

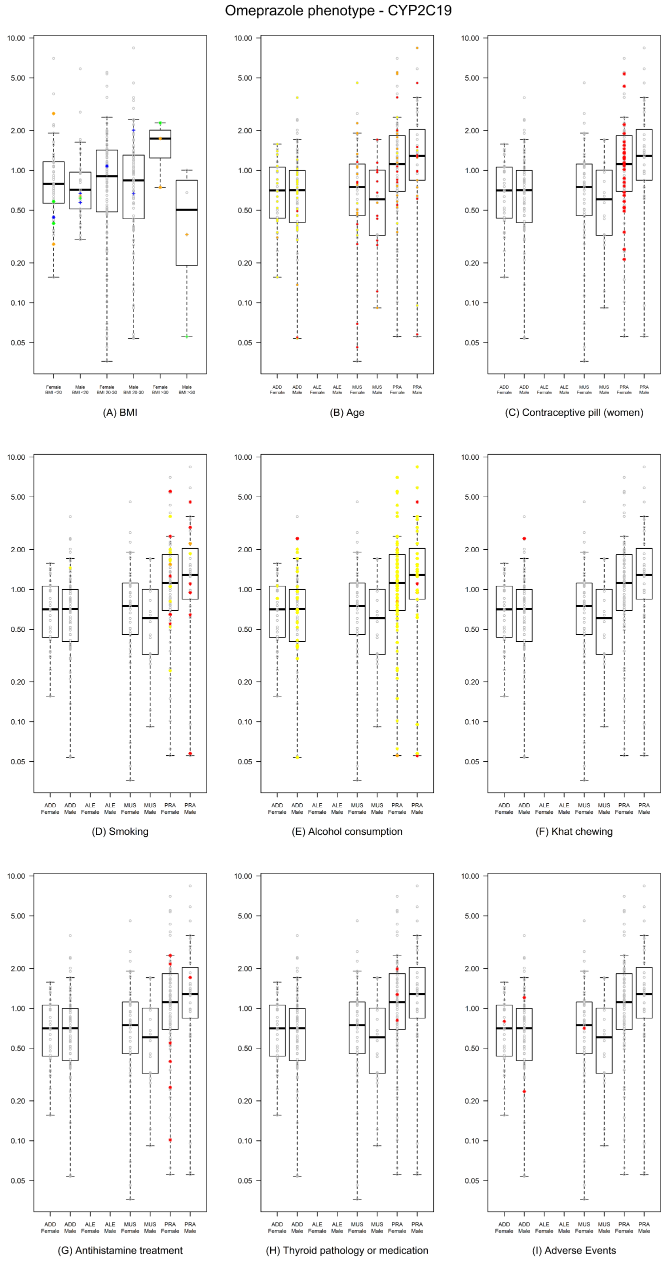

Supplementary Figure 16. Omeprazole phenotype distribution (displayed in logarithmic scale) as a function of BMI and sex (A), and of population and sex (B to I). Individual values are shown by gray circle symbols on top of boxplots. In (A) star and plus symbols indicate, respectively, individuals with BMI above or below the 95% of the BMI distribution in their sex category and population (ADD: blue, MUS: orange, ALE: red, PRA: green). In (B) star symbols indicate, respectively, individuals younger than 21 years (< the first quartile of age distribution, yellow), older than 24 years (> the third quartile of age distribution, orange), or older than 28 years (> the 85% of age distribution, red). In (C) red stars indicate women taking contraceptive pill. In (D) and (E) star symbols indicate individuals that, respectively, smoke or drink alcohol daily (red), weekly (orange) or occasionally (yellow). In (F) the red star symbol indicates the individual that chews khat. In (G), (H) and (I) red star symbols indicate, respectively, individuals with a history of antihistamine treatment, of thyroid pathology or thyroid treatment, or that experienced an adverse event during the sampling trial.

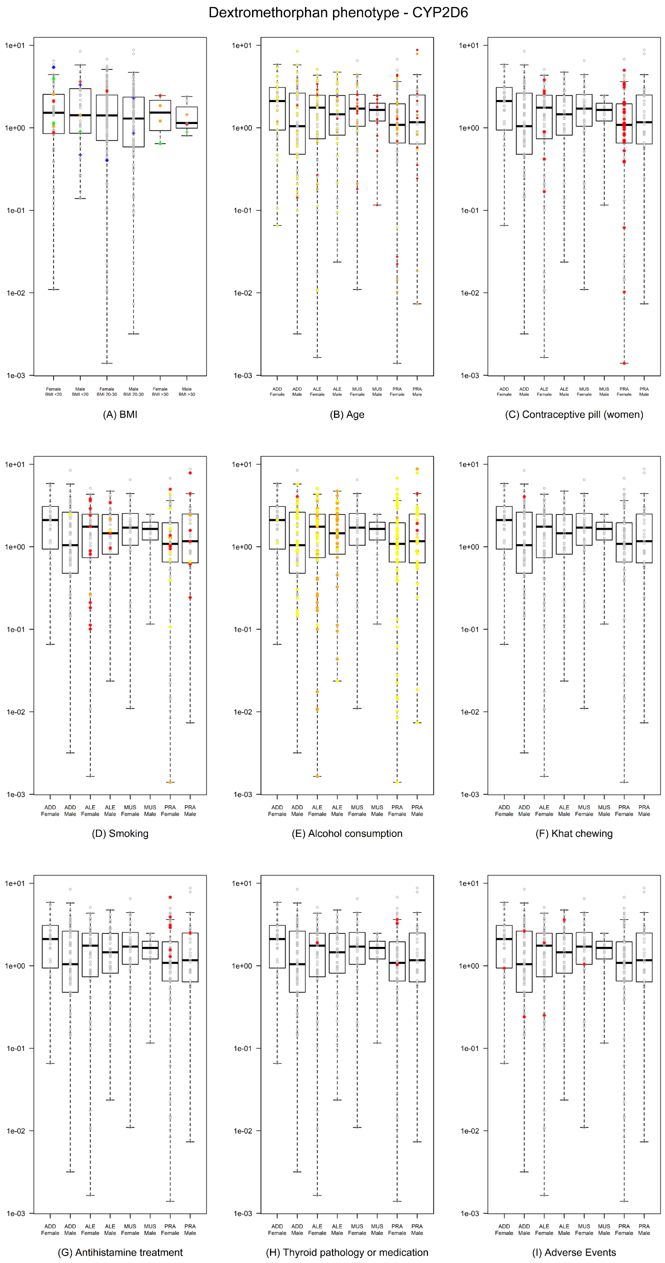

Supplementary Figure 17. Dextromethorphan phenotype distribution (displayed in logarithmic scale) as a function of BMI and sex (A), and of population and sex (B to I). Individual values are shown by gray circle symbols on top of boxplots. In (A) star and plus symbols indicate, respectively, individuals with BMI above or below the 95% of the BMI distribution in their sex category and population (ADD: blue, MUS: orange, ALE: red, PRA: green). In (B) star symbols indicate, respectively, individuals younger than 21 years (< the first quartile of age distribution, yellow), older than 24 years (> the third quartile of age distribution, orange), or older than 28 years (> the 85% of age distribution, red). In (C) red stars indicate women taking contraceptive pill. In (D) and (E) star symbols indicate individuals that, respectively, smoke or drink alcohol daily (red), weekly (orange) or occasionally (yellow). In (F) the red star symbol indicates the individual that chews khat. In (G), (H) and (I) red star symbols indicate, respectively, individuals with a history of antihistamine treatment, of thyroid pathology or thyroid treatment, or that experienced an adverse event during the sampling trial.

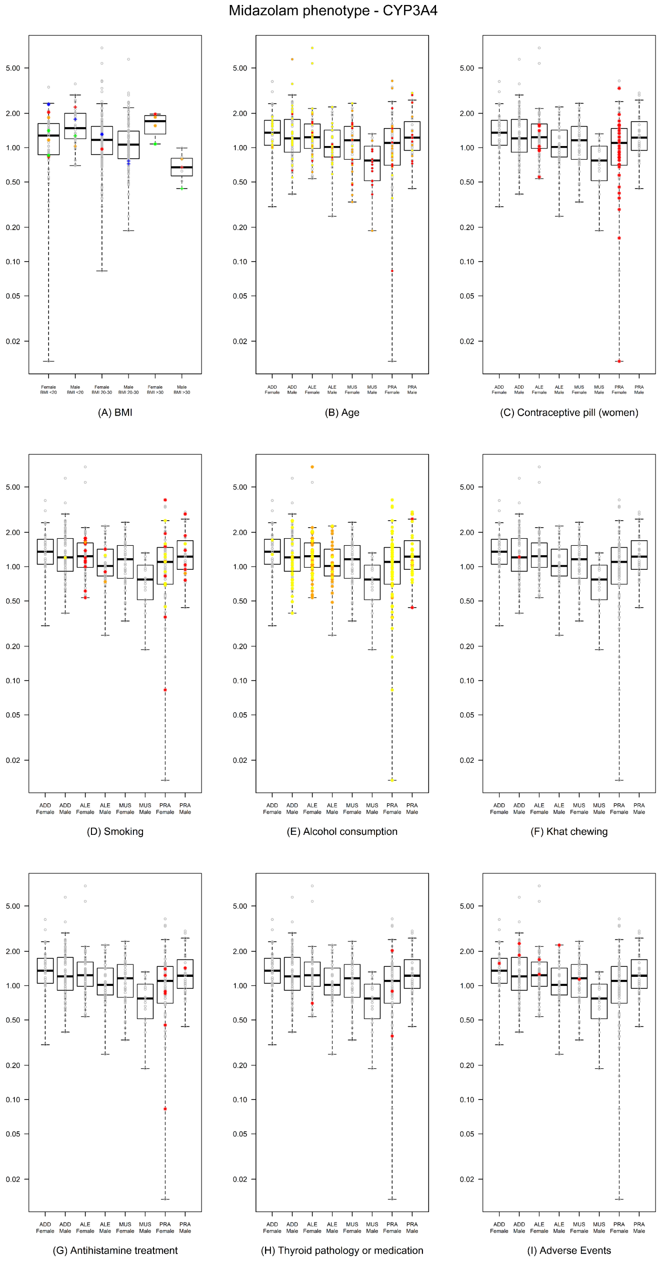

Supplementary Figure 18. Midazolam phenotype distribution (displayed in logarithmic scale) as a function of BMI and sex (A), and of population and sex (B to I). Individual values are shown by gray circle symbols on top of boxplots. In (A) star and plus symbols indicate, respectively, individuals with BMI above or below the 95% of the BMI distribution in their sex category and population (ADD: blue, MUS: orange, ALE: red, PRA: green). In (B) star symbols indicate, respectively, individuals younger than 21 years (< the first quartile of age distribution, yellow), older than 24 years (> the third quartile of age distribution, orange), or older than 28 years (> the 85% of age distribution, red). In (C) red stars indicate women taking contraceptive pill. In (D) and (E) star symbols indicate individuals that, respectively, smoke or drink alcohol daily (red), weekly (orange) or occasionally (yellow). In (F) the red star symbol indicates the individual that chews khat. In (G), (H) and (I) red star symbols indicate, respectively, individuals with a history of antihistamine treatment, of thyroid pathology or thyroid treatment, or that experienced an adverse event during the sampling trial.

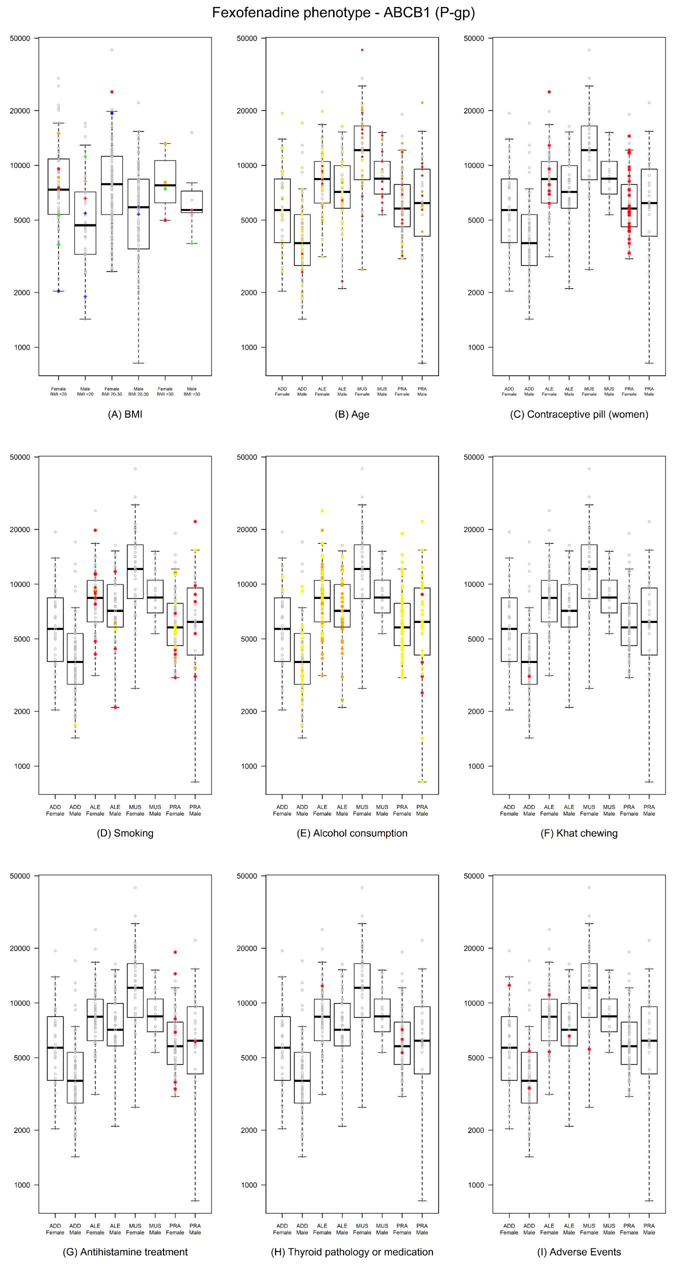

Supplementary Figure 19. Fexofenadine phenotype distribution (displayed in logarithmic scale) as a function of BMI and sex (A), and of population and sex (B to I). Individual values are shown by gray circle symbols on top of boxplots. In (A) star and plus symbols indicate, respectively, individuals with BMI above or below the 95% of the BMI distribution in their sex category and population (ADD: blue, MUS: orange, ALE: red, PRA: green). In (B) star symbols indicate, respectively, individuals younger than 21 years (< the first quartile of age distribution, yellow), older than 24 years (> the third quartile of age distribution, orange), or older than 28 years (> the 85% of age distribution, red). In (C) red stars indicate women taking contraceptive pill. In (D) and (E) star symbols indicate individuals that, respectively, smoke or drink alcohol daily (red), weekly (orange) or occasionally (yellow). In (F) the red star symbol indicates the individual that chews khat. In (G), (H) and (I) red star symbols indicate, respectively, individuals with a history of antihistamine treatment, of thyroid pathology or thyroid treatment, or that experienced an adverse event during the sampling trial.

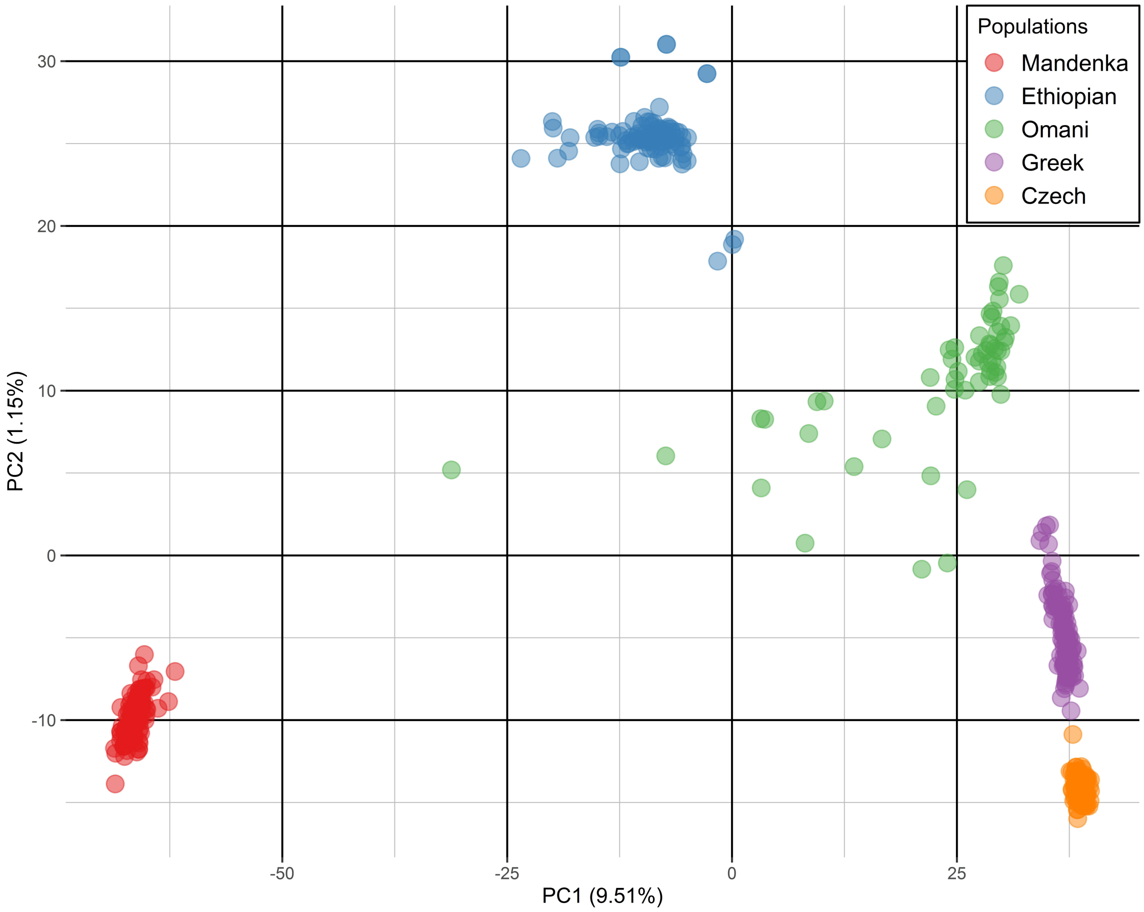

Supplementary Figure 20. Principal component analysis of the four ADME and the Senegalese Mandenka. The first and the second components account for 9.51% and 1.15% of the total variance, respectively.

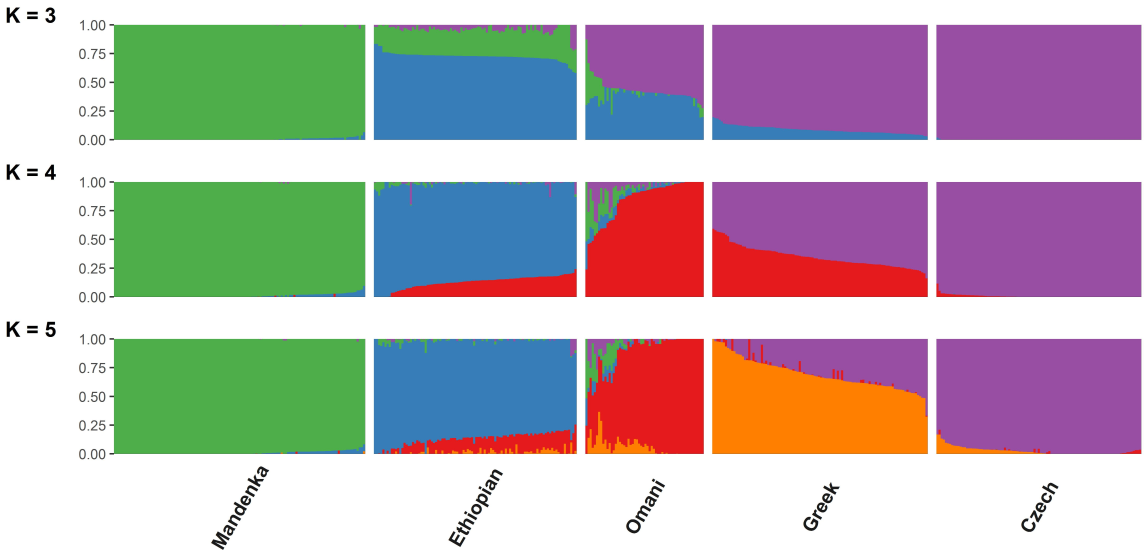

Supplementary Figure 21. ADMIXTURE plot of the four ADME and the Senegalese Mandenka for different values of K (clusters). As shown on Supplementary Figure 24, the model with the best cross-validation criterion is at K = 4.

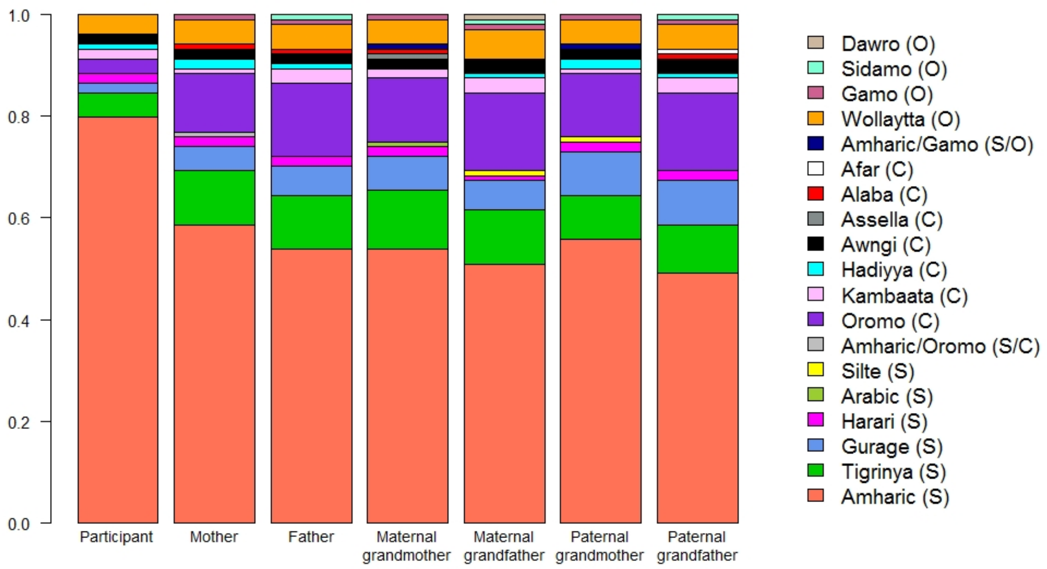

Supplementary Figure 22. Distribution of the mother tongues of the Ethiopian participants to the study and their relatives (parents and grandparents). All languages are Afro-Asiatic, from the Semitic (S), Cushitic (C) or Omotic branches (O), respectively.

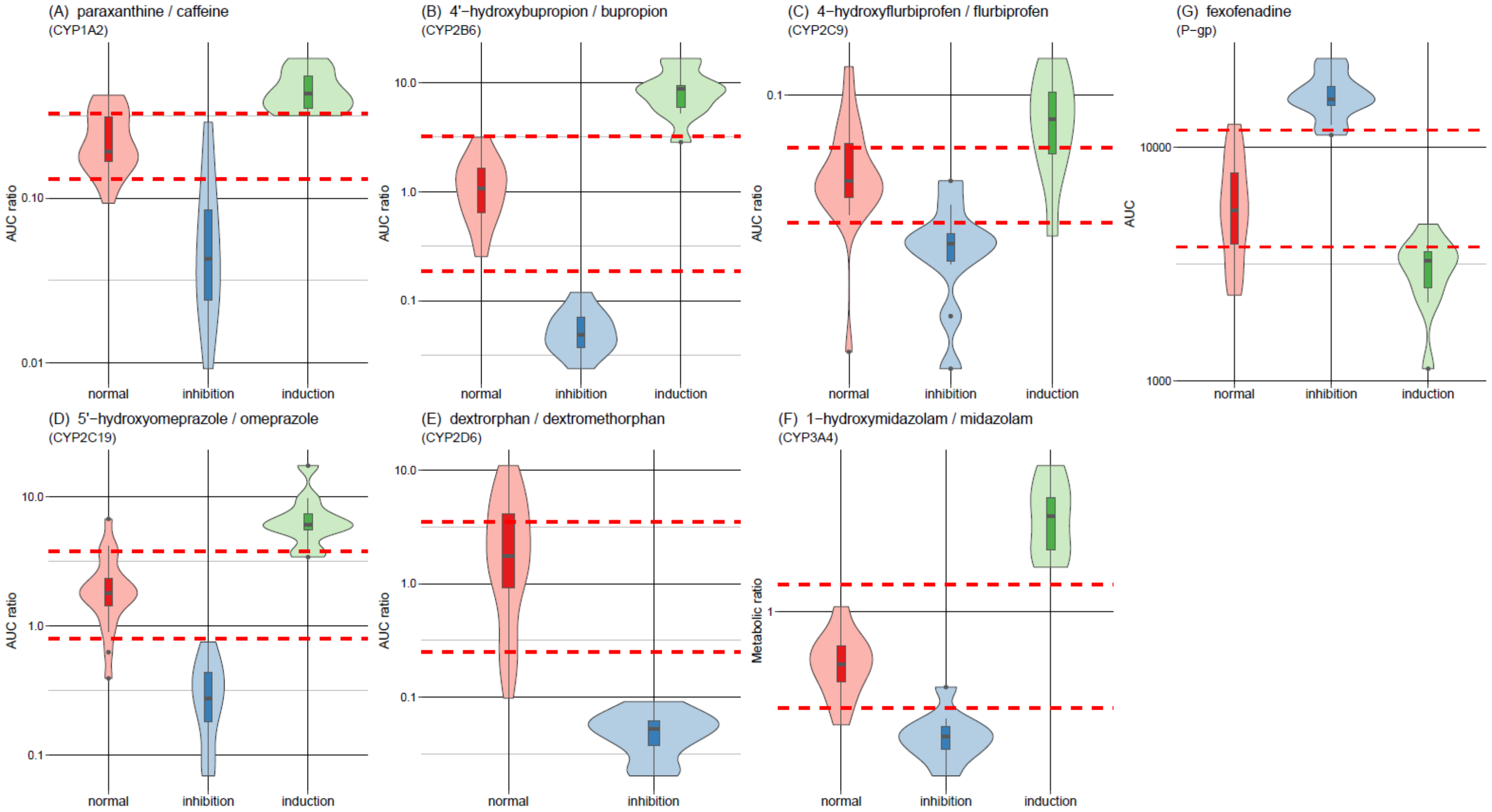

Supplementary Figure 23. Violin plots of AUC ratios, or MR ratio in the case of midazolam (CYP3A4), of the activity of the seven Geneva cocktail compounds (all displayed in logarithmic scale), measured with/without induction/inhibition in the study cohort of Bosilkovska *et al.* (1). The lower and higher thresholds set to classify phenotypes into three metabolizer categories (poor, extensive, rapid) are shown by red dotted lines.

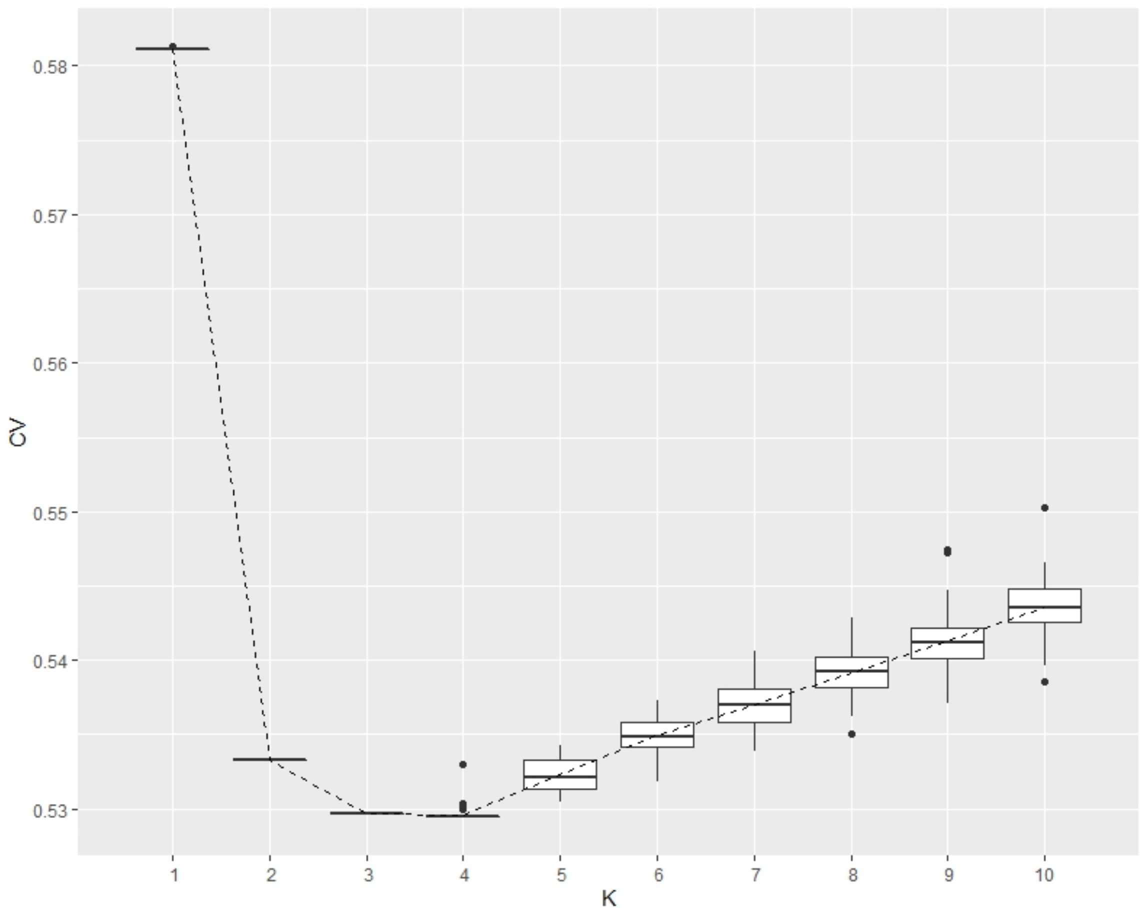

Supplementary Figure 24. Box-plots of cross-validation (CV) criteria for the different models tested with ADMIXTURE. For each value of cluster (K), 50 iterations were computed.

Supplementary references

1. M. Bosilkovska, C. F. Samer, J. Déglon, M. Rebsamen, C. Staub, P. Dayer, B. Walder, J. A. Desmeules, and Y. Daali, Geneva Cocktail for Cytochrome P450 and P-Glycoprotein Activity Assessment Using Dried Blood Spots, *Clinical Pharmacology & Therapeutics* **96**, 349–359 (2014).
2. V. Rollason, M. Mouterde, Y. Daali, M. Čížková, E. Priehodová, I. Kulichová, H. Posová, J. Petanová, A. Mulugeta, E. Makonnen, A. Al-Habsi, R. Davidson, K. K. Al-Balushi, K. Al-Thihli, M. Cerná, S. Al-Yahyaee, V. Černý, G. Yimer, E. S. Poloni, and J. Desmeules, Safety of the Geneva Cocktail, a Cytochrome P450 and P-Glycoprotein Phenotyping Cocktail, in Healthy Volunteers from Three Different Geographic Origins, *Drug Safety* **43**, 1181–1189 (2020).
3. M. Bosilkovska, J. Déglon, C. Samer, B. Walder, J. Desmeules, C. Staub, and Y. Daali, Simultaneous LC–MS/MS quantification of P-glycoprotein and cytochrome P450 probe substrates and their metabolites in DBS and plasma, *Bioanalysis* **6**, 151–164 (2014).
4. Y. Gloor, M. Mouterde, A. Matthey, E. Poloni, J. Chabert, A. Simona, L. Bovet, M. Besson, V. Piguet, C. Cedraschi, G. Pickering, E. Kosek, G. Ehret, and J. Desmeules, P192 - Identification of genetic determinants for central pain sensitization in fibromyalgia patients, Drug Metabolism and Pharmacokinetics, ABSTRACTS FROM THE 12TH INTERNATIONAL ISSX MEETING 28–31 July 2019 35, S81 (2020).
5. S. Purcell, B. Neale, K. Todd-Brown, L. Thomas, M. A. Ferreira, D. Bender, J. Maller, P. Sklar, P. I. De Bakker, M. J. Daly, et al., Plink: a tool set for whole-genome association and population-based linkage analyses, *The American Journal of Human Genetics* **81**, 559–575 (2007).
6. Y. Guo, J. He, S. Zhao, H. Wu, X. Zhong, Q. Sheng, D. C. Samuels, Y. Shyr, and J. Long, Illumina human exome genotyping array clustering and quality control, *Nature Protocols* **9**, 2643–2662 (2014).
7. 1000 Genomes Project Consortium, A global reference for human genetic variation, Nature 526, 68–74 (2015).
8. P. Danecek, A. Auton, G. Abecasis, C. A. Albers, E. Banks, M. A. DePristo, R. E. Handsaker, G. Lunter, G. T. Marth, S. T. Sherry, G. McVean, and R. Durbin, The variant call format and VCFtools, *Bioinformatics* **27**, 2156–2158 (2011).
9. T. Jombart, Adegenet: a r package for the multivariate analysis of genetic markers, *Bioinformatics* **24**, 1403–1405 (2008).
10. Alexander, D.H., Novembre, J., and Lange. K. 2009. Fast model-based estimation of ancestry in unrelated individuals. *Genome Research*, 19:1655-1664.
11. S. Das, L. Forer, S. Schönherr, C. Sidore, A. E. Locke, A. Kwong, S. I. Vrieze, E. Y. Chew, S. Levy, M. McGue, D. Schlessinger, D. Stambolian, P.-R. Loh, W. G. Iacono, A. Swaroop, L. J. Scott, F. Cucca, F. Kronenberg, M. Boehnke, G. R. Abecasis, and C. Fuchsberger, Next-generation genotype imputation service and methods, *Nature genetics* **48**, 1284–1287 (2016).
12. P.-R. Loh, P. Danecek, P. F. Palamara, C. Fuchsberger, Y. A. Reshef, H. K. Finucane, S. Schoenherr, L. Forer, S. McCarthy, G. R. Abecasis, R. Durbin, and A. L. Price, Reference-based phasing using the Haplotype Reference Consortium panel, *Nature Genetics* **48**, 1443–1448 (2016).
13. K. Watanabe, E. Taskesen, A. v. Bochoven, and D. Posthuma, Functional mapping and annotation of genetic associations with FUMA, *Nature Communications* **8**, 1–11 (2017).

14. P. C. Sabeti, P. Varilly, B. Fry, J. Lohmueller, E. Hostetter, C. Cotsapas, X. Xie, E. H. Byrne, S. A. McCarroll, R. Gaudet, S. F. Schaffner, and E. S. Lander, Genome-wide detection and characterization of positive selection in human populations, *Nature* **449**, 913–918 (2007).

15. M. Gautier, A. Klassmann, and R. Vitalis, Rehh 2.0: a reimplementation of the R package rehh to detect positive selection from haplotype structure, *Molecular Ecology Resources* **17**, 78–90 (2017).

16. Yi, X., Liang, Y., Huerta-Sanchez, E., Jin, X., Cuo, Z.X., Pool, J.E., Xu, X., Jiang, H., Vinckenbosch, N., Korneliussen, T.S., Zheng, H., Liu, T., He, W., Li, K., Luo, R., Nie, X., Wu, H., Zhao, M., Cao, H., Zou, J., Shan, Y., Li, S., Yang, Q., Asan, Ni, P., Tian, G., Xu, J., Liu, X., Jiang, T., Wu, R., Zhou, G., Tang, M., Qin, J., Wang, T., Feng, S., Li, G., Huasang, Luosang, J., Wang, W., Chen, F., Wang, Y., Zheng, X., Li, Z., Bianba, Z., Yang, G., Wang, X., Tang, S., Gao, G., Chen, Y., Luo, Z., Gusang, L., Cao, Z., Zhang, Q., Ouyang, W., Ren, X., Liang, H., Zheng, H., Huang, Y., Li, J., Bolund, L., Kristiansen, K., Li, Y., Zhang, Y., Zhang, X., Li, R., Li, S., Yang, H., Nielsen, R., Wang, J., Wang, J. 2010. Sequencing of 50 human exomes reveals adaptation to high altitude. *Science*, 329(5987):75-8.

17. Fumagalli, M., Moltke, I., Grarup, N., Racimo, F., Bjerregaard, P., Jørgensen, M.E., Korneliussen, T.S., Gerbault, P., Skotte, L., Linneberg, A., Christensen, C., Brandslund, I., Jørgensen, T., Huerta-Sánchez, E., Schmidt, E.B., Pedersen, O., Hansen, T., Albrechtsen, A., Nielsen, R. 2015. Greenlandic Inuit show genetic signatures of diet and climate adaptation. *Science*, 349(6254):1343-7.

18. Turner, S.D. 2018. qqman: an R package for visualizing GWAS results using Q-Q and manhattan plots*. Journal of Open Source Software*, 3(25):731.

19. Voight, B.F., Kudaravalli, S., Wen, X., and Pritchard, J.K. 2006. A map of recent positive selection in the human genome. PLOS Biol 4(3): e72.
